# Atomic resolution tracking of nerve-agent simulant decomposition and host metal–organic framework response in real space

**DOI:** 10.1038/s42004-020-00439-1

**Published:** 2021-01-04

**Authors:** Maxwell W. Terban, Sanjit K. Ghose, Anna M. Plonka, Diego Troya, Pavol Juhás, Robert E. Dinnebier, John J. Mahle, Wesley O. Gordon, Anatoly I. Frenkel

**Affiliations:** 1 Max Planck Institute for Solid State Research, Heisenbergstr. 1, 70569 Stuttgart, Germany; 2 National Synchrotron Light Source II, Brookhaven National Laboratory, Upton, New York, NY 11973 USA; 3 Department of Materials Science and Chemical Engineering, Stony Brook University, Stony Brook, New York, NY 11794 USA; 4 Department of Chemistry, Virginia Tech, Blacksburg, VA 24061 USA; 5 Computational Science Initiative, Brookhaven National Laboratory, Upton, New York, NY 11973 USA; 6 U.S. Army Combat Capabilities Development Command Chemical Biological Center, Aberdeen Proving Ground, MD 21010 USA; 7 Chemistry Division, Brookhaven National Laboratory, Upton, New York, NY 11973 USA

**Keywords:** Metal-organic frameworks, Porous materials

## Abstract

Gas capture and sequestration are valuable properties of metal–organic frameworks (MOFs) driving tremendous interest in their use as filtration materials for chemical warfare agents. Recently, the Zr-based MOF UiO-67 was shown to effectively adsorb and decompose the nerve-agent simulant, dimethyl methylphosphonate (DMMP). Understanding mechanisms of MOF-agent interaction is challenging due to the need to distinguish between the roles of the MOF framework and its particular sites for the activation and sequestration process. Here, we demonstrate the quantitative tracking of both framework and binding component structures using in situ X-ray total scattering measurements of UiO-67 under DMMP exposure, pair distribution function analysis, and theoretical calculations. The sorption and desorption of DMMP within the pores, association with linker-deficient Zr6 cores, and decomposition to irreversibly bound methyl methylphosphonate were directly observed and analyzed with atomic resolution.

## Abstract (web summary)

Metal–organic frameworks have been shown to adsorb and decompose chemical warfare agents, but their mechanism of action is not completely understood. Here the authors quantitatively track the binding and decomposition product structures of nerve-agent simulant dimethyl methylphosphonate in host UiO-67 through in situ X-ray total scattering measurements, pair distribution function analysis, and density functional theory calculations.

## Introduction

After two decades of extensive investigations into metal–organic framework (MOF) syntheses, properties, and functionalities^1–3^, industrial MOF-based technologies have primarily emerged in the form of gas capture products^4^. This is a broad area with applications in energy storage^5,6^, food preservation^6,7^, and gas filtration, e.g., remediation of chemical warfare agents (CWAs)^8–13^. Current technologies for the uptake and decomposition of CWAs are in need of improvements to reduce problems with re-emission, low capacity, and disposal. The further discovery of suitable materials, and synthetic optimization, both require a detailed atomistic understanding of the uptake and decomposition mechanisms^11^.

Recently, Zr-MOFs have emerged as an important group of candidate materials for the destruction of CWAs. Zr-metal centers act as Lewis active sites for the hydrolysis of organophosphonates, while frameworks built from Zr(IV)-O inorganic centers are particularly attractive due to their exceptional stability in harsh chemical and high temperature conditions. In situ experimental studies aided by theoretical calculations have identified phosphonic acid products from hydrolysis reactions and their immediate coordination environments, helping to advance our understanding of mechanisms for catalytic decontamination of CWAs^8^. Density functional theory (DFT) calculations showed that the degradation of organophosphorus molecules requires nucleophilic addition of hydroxyl groups to generate pentacoordinated phosphorus intermediates that rapidly decompose^14^. This was supported by experimental evidence from in situ X-ray powder diffraction (XRPD), extended X-ray absorption fine structure (EXAFS), and diffuse reflectance infrared Fourier transform spectroscopy (DRIFTS) on MOFs based on Zr_6_O_8_ secondary building units interlinked by different organic ligands; sorption of nerve-agent Sarin simulant, dimethyl methylphosphonate (DMMP), leads to binding and decomposition into phosphonate products by the clusters, in particular, irreversibly bidentate-bound methyl methylphosphonate (MMPA)^11^. Resulting from these extensive studies, optimized Zr-MOF catalysts have been found to neutralize nerve agents within seconds in controlled laboratory conditions under basic solutions^8^, although their performance at the solid-vapor interface is hampered by strong binding of CWA decomposition intermediates and products. Therefore, while the already established materials show potential for CWA decomposition, their optimization requires space-resolved studies of sorption and decomposition mechanisms covering broad length scales (from ångströms to nanometers), and therefore, combining experimental probes with in situ capability and atomic-level sensitivity.

Various techniques have been used to examine and track the structuring of solvent/gas species and adsorbate–adsorbent interactions within porous media, in both ex situ and in situ modes. This has primarily focused on identifying electron density associated with guest molecules or binding sites, typically by single crystal or powder diffraction, using X-rays or neutrons^15–18^. However, molecular-level resolution of these features relies on an ordered nature of both host and guest sites, due to averaging over unit cells, which is often not satisfied. Modeling methods such as SQEEZE^19^, the maximum entropy method (MEM)^20^, or the use of difference envelope densities (DED)^21,22^, can help to localize the electron density in less ordered cases, but further suffer in resolution by averaging effects and cannot typically elucidate the specific nature of the interactions. On the other hand, spectroscopic techniques such as solid-state nuclear magnetic resonance SSNMR^23^, infrared spectroscopy^24^, or inelastic neutron (or X-ray) scattering^25–27^ can be used to measure and track the chemical or dynamical nature of guest molecules and their interactions during adsorption or reaction processes. However, while providing a powerful reference for theoretical predictions, they do not typically allow for direct refinement of physical models. In situ EXAFS provides a means to track the local structure directly, but is inherently element-specific, and only observable over the first few coordination shells^28,29^.

Pair distribution function (PDF) analysis can bridge the gap in length scales from an average unit cell or pore structures (nanometers) to sub-Å resolution of localized atomic coordination environments. PDF analysis uses all coherent scattering information (Bragg and diffuse) to obtain a fingerprint of real-space, atom-pair distances, regardless of the state of ordering^30–32^. The method is well established for assessing structural details of inorganic centers and defects in non-ideal MOFs^33^. It has previously been used to characterize local structuring and microporosity in amorphous and nanocrystalline MOFs^34,35^, nucleation of precursors during MOF synthesis, including the isoreticular UiO-66^36,37^, and the study of temperature-driven local distortions of Zr_6_O_8_ clusters in related MOFs UiO-66 and NU-1000^38^. It has also been used to probe the sub-structuring of both ions^39–41^ and gases^42–44^ within microporous compounds including the binding of various species to Zr_6_O_8_ clusters^45–48^. Here, we show that it can be further applied for tracking reactive changes in these guest sub-structures.

In the current study, we augment our understanding of the mechanism proposed for the uptake and decomposition of DMMP within the MOF UiO-67 [Zr_6_(μ_3_-O)_4_(μ_3_-OH)_4_(BPDC)_6_; BPDC: biphenyl-4,4′-dicarboxylate]. We demonstrate PDF analysis of in situ total scattering data to track, simultaneously, with high real-space resolution, the uptake, binding, and decomposition of the CWA simulant DMMP and associated changes in the host-framework structure through steps from framework activation to gas adsorption–desorption and regeneration. We thereby provide recipes for applying this method toward identifying and tracking reactive changes of weakly scattering components within a host structure. Such studies will be valuable for furthering our understanding of the physical characteristics underpinning sorption and reaction capacities for targeted materials design. The study of functional mechanisms in these materials may become increasingly important as new synthetic techniques are being rapidly developed for cheap, environmentally friendly, and scalable syntheses^49^.

## Results

### Average structure and thermal behavior

**Fig. 1:**
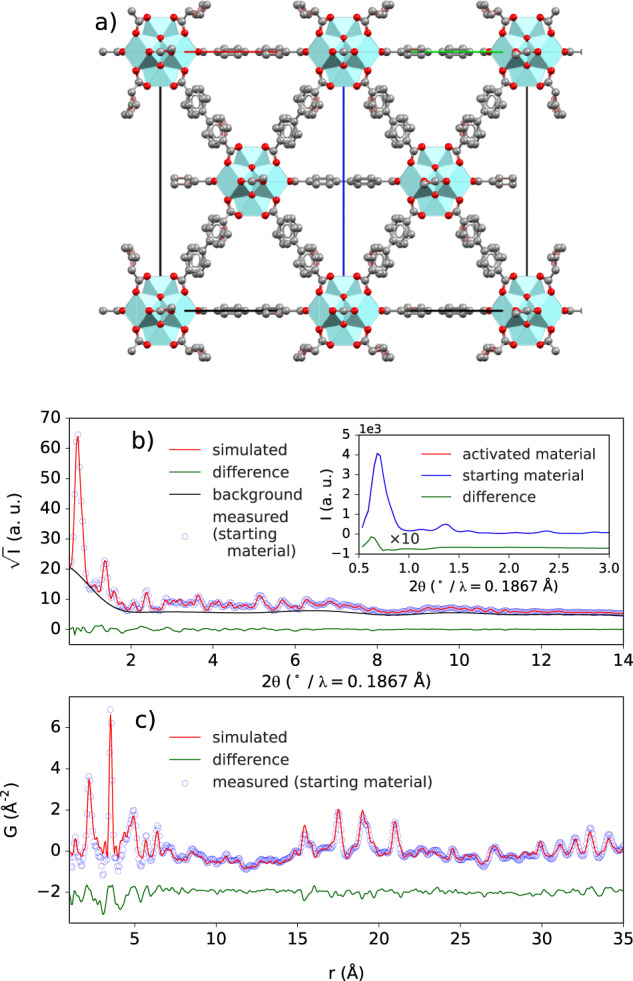
UiO-67 structure refinements. **a** The Zr_6_O_8_ clusters coordinated by 12 BPDC ligands in a 3D network with *F*432 space group (along the [110] direction). **b** Resulting fit from Rietveld refinement (*R*_w_ = 5.18%) for activated UiO-67. The inset shows a comparison of the diffraction patterns before and after activation and suggests only a slight change in relative intensity of the first Bragg peak which contains contributions from 111 and 200. **c** Fit from real-space structure refinement (*R*_w_ = 26%) to the measured PDF of activated UiO-67.

The structure of UiO-67 is reported with space group symmetry $Fm{\overline{3}}m$, with occupational disorder on oxygen positions in the Zr_6_O_8_ clusters as well as on the phenyl rings in the biphenyl-4,4′-dicarboxylic acid (BPDC) groups^50^. Here, a structure with space group symmetry *F*432 was used to allow for the single occupation of biphenyl groups with the proper torsion angle, i.e., ~30°^51^. The doubled oxygen sites in the cluster were also removed, in order to remove pair correlations coming from between the same atom sitting on nearby disordered sites. Rietveld refinements of the crystal structures with both $Fm{\overline{3}}m$ and F432 symmetries were tested on the data collected from the UiO-67 starting material, prior to activation. Both structures gave a satisfactory fit. The *F*432 structure refinement resulted in larger atomic displacement parameters (ADPs), presumably due to the lack of conformationally disordered phenyl rings, but resulted in a slightly better *R*_w_ = 5.18% versus 6.11% for $Fm{\overline{3}}m$. The *F*432 structure, and results of associated Rietveld and real-space PDF refinements, are shown in Fig. 1. Structure refinements to data from UiO-67 at RT, before and after activation, resulted in the agreement of the lattice parameter and showed that there was not a significant structural modification due to activation. There was a small but discernable increase in the intensity of the 111 peak, Fig. 1b, inset, which can be associated with the removal of pore content. Importantly, both Rietveld and PDF refinements were improved by allowing for BPDC vacancies, as has been widely reported for Zr-based MOFs^52–55^. Here, we observe ~1−3 vacancies per node via Rietveld or PDF refinement respectively.

During activation under He, the lattice parameter contracted by ~0.01 Å (~0.04%), or ~0.1% decrease in volume. This was initially attributed to the removal of guest solvent molecules. However, on returning to RT, the lattice parameter also returned to the starting value of 26.85(3) Å (PDF). To check reproducibility, the activated UiO-67 was reheated to 80 °C in 5 °C steps, which showed incremental and systematic contraction, indicating that there is a slight isotropic negative thermal expansion (NTE) of UiO-67, at least over the range of RT to 80 °C. Representative lattice parameter values from Rietveld and PDF refinement are given in Supplementary Table 1. Using the slope of the volumes determined during the reheating step III, a volumetric NTE coefficient of *α*_V_ = −10.78(8) MK^−1^ was determined over ~30–80 °C. To the best of our knowledge, NTE in UiO-67 has not been reported, perhaps because it is weak. This effect is fairly common in MOFs, and can be very large, e.g., UiO-66(Hf)^56^. NTE is expected to correlate with linker defects (e.g., vacancies) which can decrease the stiffness of the framework. However, it was found to be anti-correlated with defect concentration, which could be partially explained by the densification of the MOF with increased defect concentration^56^. The dynamics of BPDC could also have an effect^51^.

### Adsorption and desorption of DMMP

**Fig. 2:**
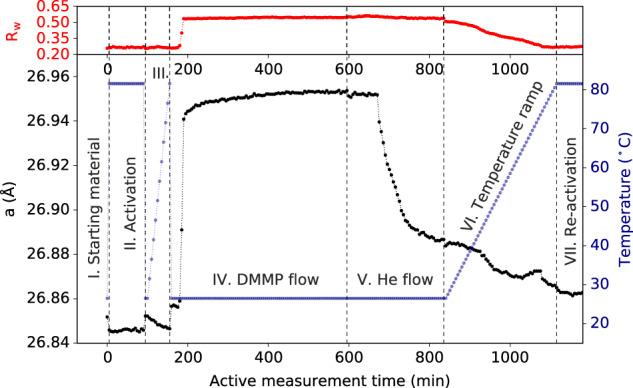
Lattice parameters from refinement. Lattice parameter *a* (black dots, left axis) during steps I–VII with respect to active measurement time (5 min increments: only continuous within a given experimental step). Dashed vertical lines represent time breaks between steps. The temperature (blue dots, right axis) and goodness-of-fit *R*_w_ from real-space structure refinement (red dots, top) are given for every data point.

We turn our attention to the structural changes that occur in the UiO-67 framework during DMMP sorption and desorption steps. Structure refinements to the PDF data were performed for all steps of the experiment to track changes in both long-range order (LRO) and short-range order (SRO). Changes in the LRO primarily consist of variations in the lattice parameter and in the distribution of atomic density. The lattice parameters from PDF refinement are plotted as a function of the “active measurement time”, wherein the time refers to the fact that each data point represents a 5 min measurement, Fig. 2. This time is only continuous within a given measurement step, and time gaps between stages of the experiment are not included. After activation (step II) and reheating (step III), the temperature was returned to RT, and step IV began with the DMMP vapor/He mixture introduced to the flow cell at RT. The lattice parameter increased slightly within the first 5 min (length of the first measurement), indicating that some volume of DMMP quickly entered the framework. There was a delay in further expansion until ~25 min, when *a* increased rapidly over the next 10 min, and then continued to increase gradually for the rest of the exposure time until saturating at around *a ~* 26.95(7) Å (0.376% increase), or ~1.14% increase in volume, in good agreement with the volumetric increase of 1.17% previously reported^11^. This shows that the guest molecules exert an internal expansive strain on the framework, which is opposite and much larger in magnitude than the NTE effects due to increasing temperature over the range studied. The loading of DMMP gas is further supported by the systematic decrease in relative intensity of the 111 and 200 Bragg reflections, which is characteristic of the decrease in scattering contrast between pore and framework that occurs on guest loading (see Supplementary Figs. 2 and 3). On switching the DMMP vapor/He mixture to just He at RT, step V, there was a slight decrease in the lattice parameter. It is unclear if this was due to an immediate exchange of a small amount of DMMP for He, or to the loss of some DMMP between active measurement. Either way, the lattice parameter remained constant for ~75 min, after which, the effects of exchanging DMMP for He on the lattice expansion become visible in the data. After 75–80 min, the exchange of some DMMP for He resulted in a reduction of internal strain on the framework and a contraction of the lattice. The lattice continued to contract with further exposure, though slower than the expansion step, indicating that the exchange of saturated DMMP for He is a slower process than the uptake of DMMP by activated UiO-67. The exchange continued for steps VI and VII, but with a decreasing rate.

On step VI, the He flow was continued, and the material was incrementally heated back up to 80 °C in steps of 1 °C. During this heating process, the exchange behavior becomes more complicated, likely due to the increasing temperature. We observe that at temperatures close to RT, the removal of DMMP continues in a similar fashion to the exchange with He at RT. However, as temperature increases, gas removal increases at an increasing rate until plateauing again around 55 °C. There is another sharp decrease in lattice parameter starting around 70 °C. We hypothesize that, at lower temperatures, gas in the bulk of the pores may be removed, whereas, in the higher temperature regime, sufficient energy is provided to more efficiently remove molecules that interact more strongly with the framework or other adsorbed layers. Shortly after reaching 80 °C, the lattice parameter equilibrated at 26.86(3) Å, which is larger than the activated state, indicating not all content was removed by the thermal treatment.

**Fig. 3:**
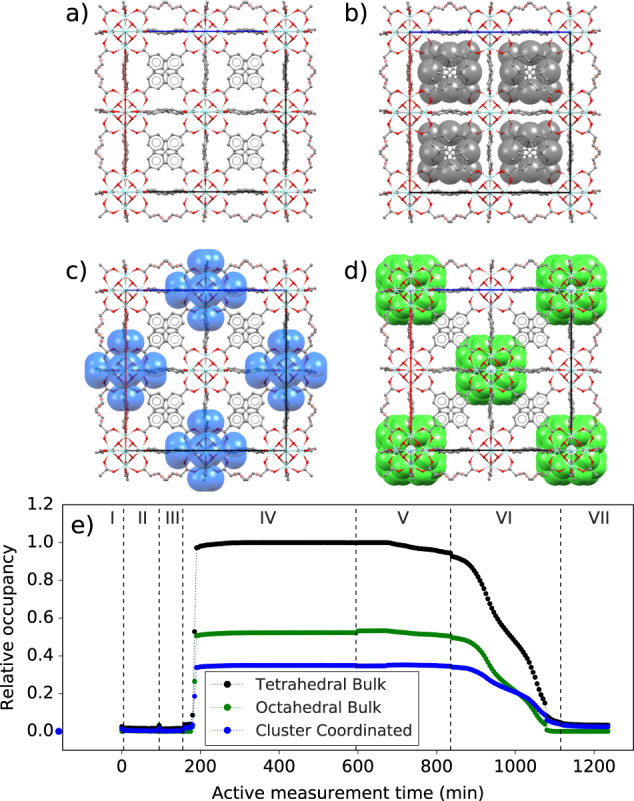
Pore site occupancies. The UiO-67 framework is shown **a** empty, **b** with filling sites in the tetrahedral pores, **c** with sites directly coordinating the Zr_6_O_8_ clusters, and **d** with filling sites in the octahedral pores. **e** Occupancies obtained from sequential Rietveld refinement are shown, relative to the maximum value of the tetrahedral occupancy.

To quantify the gas loading within the framework, the structure with *F432* symmetry was refined to the diffraction pattern of the activated sample. Then, all parameters were fixed except for the lattice parameter, scale, and background, and the model was applied to the diffraction pattern of the sample with maximum gas loading at the end of step IV. Pseudo-atoms^57^ consisting of Dy (isoelectronic with DMMP), with a large ADP, were introduced and allowed to freely adjust their positions and occupancies by simulated annealing^58^ until all changes in Bragg peak intensities could be described. This resulted in three different positions (each giving 96 sites in the used symmetry) which could be distinctly identified as corresponding to electron density in tetrahedral pores, octahedral pores, and coordinating the Zr_6_O_8_ clusters, as shown in Fig. 3a–d. Simulated annealing was turned off, and a final relaxation was run, also allowing the pseudo-atom ADP to refine. Finally, the site positions and ADP were fixed, and the model was refined to all diffraction patterns, allowing only the lattice parameter, background, scale, and pseudo-atom site occupancies to refine.

Sequential Rietveld refinements gave the same trends in lattice parameter (Supplementary Fig. 4). The values are slightly shifted, likely due to the extremely broadened peaks of the total scattering measurement. The relative occupancies for each site are plotted for every step in Fig. 3e. At maximum filling, about half as much DMMP occupies octahedral pore bulk versus tetrahedral pore bulk in the unit cell, and about a third as much directly coordinates the clusters. Interestingly, only a small amount of DMMP is removed from octahedral and tetrahedral pores during step V. This is supported by the slight increase in relative peak intensity in the diffraction patterns (Supplementary Fig. 2b), despite the significant drop in lattice parameter, and may warrant further investigation into the relationship between pore content and induced strain. The removal is significantly increased on heating, where we again see the step in removal at higher temperatures. The rate of removal of DMMP coordinating the clusters is smaller, indicating stronger interactions, and after re-activation, there is still some residual electron density in the tetrahedral and cluster coordinating sites, which likely correspond to bound states that are not removed by activation at 80 °C, supporting the larger lattice parameter at the end of the experiment. The preference for gas loading in tetrahedral pores has been predicted before for different hydrocarbons in UiO-67 and ascribed to stronger adsorption forces imposed by the tetrahedral versus octahedral cage^59^. That guest molecules tend to reside in the smallest pores accessible to them has also been noted for instance in Cu_3_(BTC)_2_^16^.

Separate thermogravimetric experiments were performed, as described in Supplementary Section 9, to gain additional insight into the adsorption and desorption of DMMP. In particular, the adsorption of DMMP into the framework and subsequent removal by heating could be corroborated, as observed by the X-ray measurements. No distinct boundary between the desorption of unreacted DMMP and/or DMMP decomposition products was detected, though all adsorbed content appeared to be removed by 120 °C.

### Local structure

**Fig. 4:**
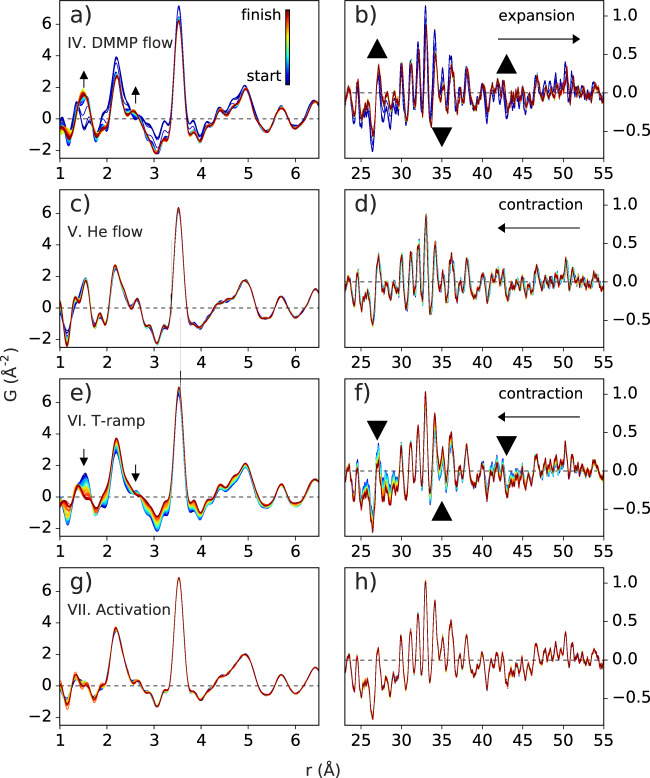
Changes in the local structure. PDFs are shown over short and long distances during steps IV: DMMP flow **a**, **b**, V: He flow **c**, **d**, VI: temperature ramp **e**, **f**, and VII: re-activation **g**, **h**. The order is start of step (blue) to end (red). Arrows mark the direction of significant changes in pair correlations or correlated density, and lattice expansion/contraction.

Real-space structure refinement to the starting material PDF gives an *R*_w_ = 0.26. This is generally considered suitable for complex organic and hybrid materials, and furthermore describes all the features in the observed signal. However, during the gas sorption steps, the *R*_w_ increases to about 0.56, indicating a progressively poorer fit to the SRO, despite maintaining a reasonably good fit to the LRO. It then decreases during desorption, eventually returning to the starting value at the end of the final activation step. Such changes could come about by signals from guest molecules and their interactions with each other and the framework and/or local distortions of framework components. To investigate this, we monitored the short-distance signals in the measured PDFs, shown in Fig. 4. The sequentially measured PDFs are superimposed, for each separate step, over a range of 1.0–6.5 Å in the left panels to highlight changes in local atom-pair bond distribution. Corresponding changes in the LRO are shown in the right panels. For step IV, changes associated with DMMP loading are observed, i.e., new atom-pair distances appear at ~1.5 and 2.6 Å and grow with time. There is also a noticeable change in atomic density as can be seen by the change in the slope of the baseline. Over long distances, a systematic shift in sharp peaks to longer distances directly shows the framework expansion as measured by the change in lattice parameter. This is accompanied by a flattening of the lowest frequency oscillation, (indicated in the direction of the large black arrows), which comes from the decrease in 111/200 Bragg peak intensities, and indicates an increase in the homogeneity of the electron density as molecules fill the pores^35,41^.

During step V, a systematic shift of the peak positions at long distances directly shows the re-contraction of the framework as some DMMP is exchanged. However, there is not a large change in the low-frequency components, indicating that the overall density distribution is not significantly modulated by the gas exchange. The signal at short distances barely changes, despite the fact that some DMMP must be exchanged due to the lattice contraction. Therefore, we conclude that He only replaces a small amount of DMMP at RT, supported by the Rietveld refinements, most likely from the pore bulk and only weakly adsorbed to the MOF or other adsorbed layers. The loss of a small amount of DMMP is further supported by a slight reduction in the peak intensity at ~1.5 Å relative to the C–C nearest neighbor (NN) peak at ~1.4 Å. During the reheating of the sample, in step VI, the lattice continues to contract; the amplitude of the pore-to-framework density modulation increases, and the unreacted guest signal goes away, until reaching the final re-activation step VI at 80 °C. Upon equilibration, the signal remains relatively constant over both short and long ranges.

### Tracking the binding behavior

**Fig. 5:**
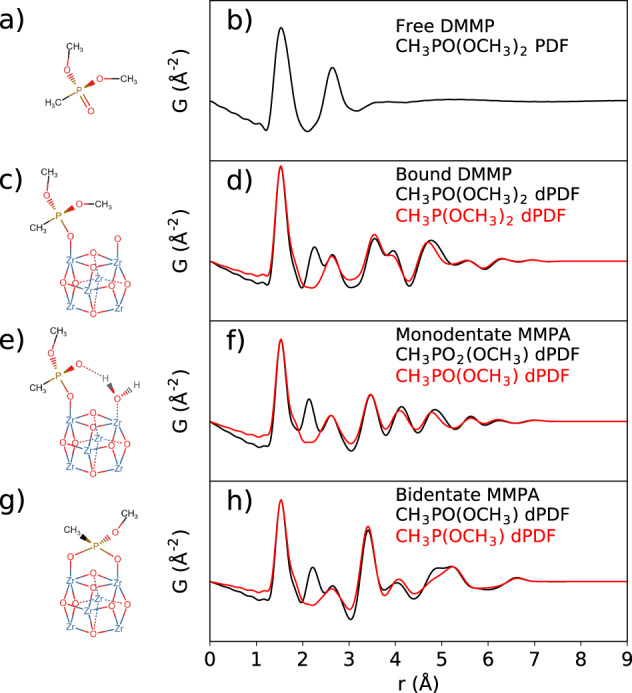
DFT structures and corresponding difference PDFs. Schematic representations and simulated PDF for isolated DMMP molecule **a**, **b**, and difference PDFs (dPDFs) representing DMMP + DMMP-cluster pair distances, for **c**, **d** bound DMMP, **e**, **f** monodentate MMPA, and **g**, **h** bidentate-bound MMPA. Red curves show the dPDFs with bridging oxygen considered as part of the cluster.

To ascertain what signals could be expected during the uptake of DMMP, models were built and relaxed by DFT. Guided by the expected reaction mechanism^60,61^, different models were prepared including isolated DMMP, DMMP bound to Zr_6_O_8_ through monodentate oxygen, and MMPA bound to Zr_6_O_8_ by either monodentate oxygen or bidentate bridging oxygen bonds as the final decomposed states after release of MeOH. For a full list of models, see Supplementary Scheme 1 and Supplementary Fig. 5. These structures are provided in Supplementary Data 1. The different interactions are depicted in Fig. 5a, c, e, and g, respectively, and corresponding, simulated PDF signals are plotted on the right. Figure 5b shows the full simulated PDF for an isolated molecule of DMMP. This is the real-space signal that can be expected for DMMP gas molecules that are not bound to any framework atoms or strongly interacting with each other. In Fig. 5d, f, and h, the difference PDFs (dPDFs) are plotted, meaning that they only include atom-pairs where at least one, or both, atoms are located in the DMMP/MMPA component. Atom-pairs, where both atoms are located in the Zr_6_O_8_ cluster, are not included since this component is not being added or subtracted during the experiment. In cases where the host structure is significantly modified, it is important to additionally account for this in extracting and interpreting the difference. We included simulations where the bridging oxygen was considered either as part of the DMMP molecule or as part of the cluster, which could respectively correspond to cases where DMMP binds to a bare cluster surface versus exchanging some prior existing surface species such as water.

Contributions from between the linkers and DMMP/MMPA were not included, since the DFT models considered the terephthalate linkers of UiO-66 rather than BPDC of UiO-67. We also do not expect strong structural correlations between guest molecules and BPDC in the PDF measurement. Notable differences occur between different binding states. For all bound states, the O–O pair distances split, reducing the intensity concentrated in the second peak of the isolated molecule. Otherwise, the dPDFs of both monodentate DMMP and MMPA are very similar with only a slight redistribution of pair distances. Binding of MMPA is apparently stronger, as the P–Zr distance decreases from 3.565 Å for DMMP to 3.502 Å for monodentate MMPA. The bidentate MMPA state shows a more distinct increase in the intensity of the P–Zr peak, due to the increased multiplicity, now at 3.412(3) Å between the two pairs.

**Fig. 6:**
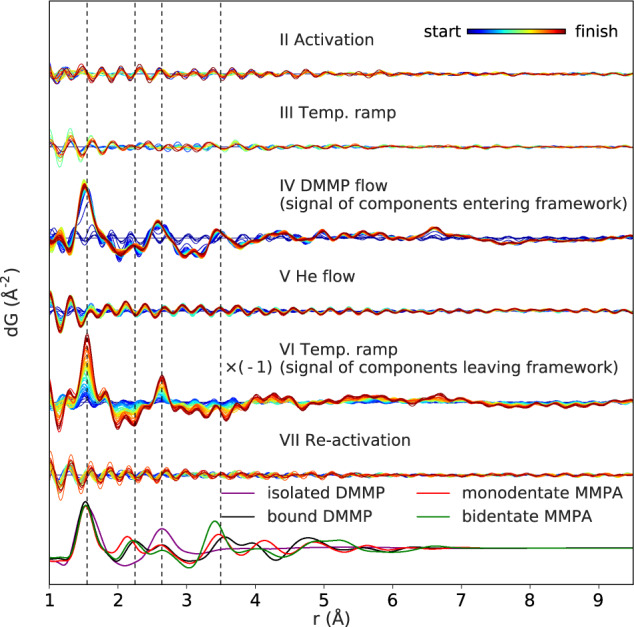
Difference PDFs for each experimental stage. The dPDFs are shown for each experimental step, showing the differential change in pair distance distribution with respect to start of a given step, and with each step offset for comparison. For step VI, the dPDFs are flipped upside-down to show the signal of components leaving the framework during heating. The simulated PDF for an isolated molecule of DMMP and the dPDFs of DMMP and MMPA binding states are plotted for reference.

To directly probe the experimental data for the signals predicted from the DFT models, dPDFs were produced for each experimental step by fitting the experimental PDF from the start of a given step, plus a low-frequency sine wave to account for the modulations in the density, to each subsequent dataset. Thus, the dPDF signal contains the atom-pair distribution not accounted for by the original framework structure, i.e., the pair distances associated with the DMMP/MMPA molecules as well as any interactions with the Zr_6_O_8_. The dPDFs for each separate experimental step are compared in Fig. 6. Despite the NTE behavior, the changes in SRO are negligible during the activation and heating steps. Likewise, the SRO changes are also negligible during step V, since only a small amount of DMMP is exchanged. Two steps show considerable changes; one is step IV during exposure to DMMP flow. Peaks in the dPDF grow at 1.5, 2.6, and 3.5 Å. The positions and relative intensities of the first two peaks indicate that most of this signal comes from free DMMP, as should be expected from previous lattice parameter and site occupation analysis. However, the increase in the intensity of the peak at 3.5 Å, which is not accounted for by the original intensities due to the Zr–Zr distances, is direct evidence for some binding resulting in correlated P–Zr pairs. The second step that shows distinct changes in the dPDFs is step VI during the temperature ramp, which removes DMMP from the pores. The dPDFs for this step have been flipped upside-down to show the increase in the signal of components leaving, rather than entering, the framework. Now, the increase in peaks at 1.5 and 2.6 Å indicate free DMMP is removed, but no feature is observed at 3.5 Å this time. Thus, bound molecules remain in the framework. If the dPDFs are instead obtained in reference to the same starting PDF of the activated sample, we observe even more detail in the changing features (Supplementary Fig. 6). An additional feature was observed to appear at ~6.68 Å during DMMP dosing, which then remains even through re-activation. As the appearance correlates with that of the peak at 3.5 Å, this could potentially be indexed to the distance between P and the 3rd NN Zr atoms at the opposite side of the cluster.

Structure-independent and structure-dependent models were used to fit the full PDFs. The first model consisted of the reference PDF of the activated state, the sine wave for the density modulation, plus three Gaussian peaks (Supplementary Section 6). An example fit is shown in Supplementary Fig. 7. The results show that the positions of the first two peaks remain relatively constant, and that the intensities at both positions drop significantly during heating in step VI, i.e., free DMMP, while the intensity of the third peak remains relatively constant, i.e., quick and non-reversible saturation of open binding sites (Supplementary Figs. 8–10). Additionally, we observe that the position of the third peak decreases throughout the experiment, from about ~3.5 to ~3.39 Å. This range is in good agreement with the range of 3.502–3.412(3) Å for the P–Zr distances observed from DFT results of monodentate and bidentate MMPA. It may therefore suggest that all bound DMMP rapidly degrades to MMPA, and that there is a distribution of binding states in MMPA that all relax toward the bidentate state. It also suggests that the re-activation step promotes this relaxation.

**Fig. 7:**
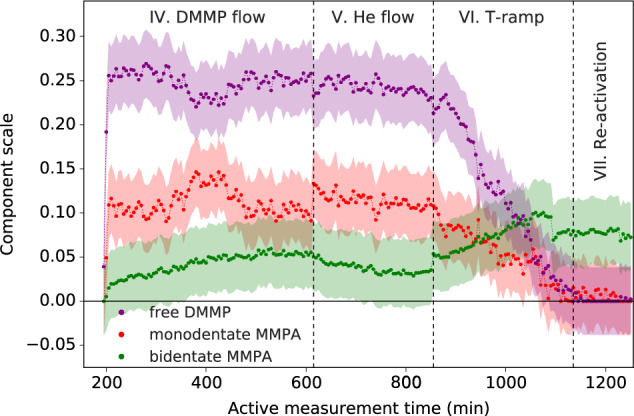
Relative binding component scale factors. Component scale factors resulting from refinement of the second model using simulated PDFs of free DMMP, monodentate MMPA, and bidentate MMPA from DFT calculations. These values do not represent atomic fractions of the respective states and should be assessed qualitatively as trends in ability of simulated signals to best describe the signals not accounted for by the starting framework. Errors are plotted as 3× the average e.s.d. from the refinement.

A second model (Supplementary Section 7) was fit to the data to quantify the relative amounts of DMMP or MMPA in different free or bound states. The same reference model was used with the PDF from the activated sample, but this time the guest components were directly accounted for using the dPDFs simulated from the different free and bound states in Fig. 5 with refined scale factors, instead of the free Gaussian peaks. Prior to refinement, the simulated dPDFs were normalized by the NN coordination shell of either DMMP or MMPA component. Example fits are shown in Supplementary Figs. 11–12. The results, shown in Fig. 7, should be taken for the general trend rather than absolute values, as the signal for discerning these components is small, and a meaningful normalization of all input components is difficult. This model further supports that the majority of DMMP is unbound in the pore bulk and is removed on heating. Both the monodentate and bidentate-bound MMPA states are preferred over bound DMMP, which was found to be negligible (Supplementary Fig. 13). Since the monodentate DMMP and MMPA states determined from DFT give very similar signals, it is possible that the monodentate MMPA signal preferred in the refinement could also encode signals from DMMP coordinating the cluster in a similar manner. Importantly, the peak assigned to P–Zr seems to be the defining feature of the bound states, and we again see a transition in preference from more monodentate-like binding to shorter, more bidentate-like binding, especially during heating. Overall, the majority of the signal observed in the dPDFs is attributed to free DMMP absorbed by the framework, while the final amount of bound MMPA was relatively small, which may be due to a low level of linker vacancies in the sample limiting the number of binding sites. We note that the modeling here is by no means exhaustive. Improved models for this analysis could consider more distributional properties of the local states, however, measurement of a sample with a higher level of binding could be beneficial for this purpose.

### Distortions of the cluster structure

**Fig. 8:**
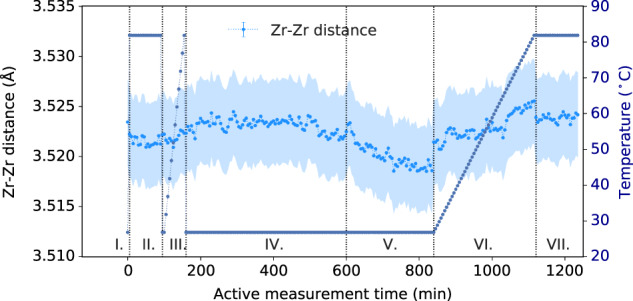
Zr–Zr nearest neighbor (NN) distances. Distances are averaged over two different fitting methods. Errors are plotted as the e.s.d.’s between the two different refinement methods (reference model versus structure refinement).

The data were also tested for distortions of the Zr_6_O_8_ clusters. Changes in the Zr–Zr NN distance were tracked by three methods: by a single Gaussian fit to the peak at 3.4–3.7 Å, by the expansion factor refined for the reference PDF for the fits shown in Fig. 7, and from the distances obtained from refinement of the structure with *F*432 symmetry to the PDF over a range of 1.0–35.0 Å. The free Gaussian fit was unreliable due to underestimated distances during loading from the unaccounted-for intensity of the P–Zr contribution, thus Fig. 8 shows the results of the latter two methods.

Guided by previous, qualitative interpretation of combined XRPD-EXAFS results^11^, distortions of the Zr–Zr distance are expected to occur by either strain in the framework induced by pore content, or by direct binding interactions. The Zr–Zr distance averaged over all DFT models of pristine, defect, and bound state models give a value of 3.53(2) Å, which is in good agreement with the range of values determined from the experiment, ~3.521(4) Å. DFT calculations found that the Zr_6_ core should anisotropically distort slightly on the exchange of an aqua ligand in the unreacted MOF for DMMP, resulting in a broadening of the NN Zr–Zr distance distribution and an expansion of the average distance by ~0.003 Å, however, the magnitude of these distortions would be too small to observe by the current experiment (Supplementary Fig. 14). On decomposition to bidentate MMPA, the NN Zr–Zr distance distribution returned to that observed for water binding. Both DFT simulations and the experimental data suggest that binding of DMMP/MMPA to Zr_6_O_8_ does not cause strong enough distortions of the clusters to generate distinctly different bond distances in the cluster, which were observed previously for higher temperature transitions^38^ or binding of NiO_x_H_y_^47^.

Since the Zr–Zr peak is averaged over all clusters, it, therefore, includes pairs in clusters unaffected by binding, making it difficult to separate out binding-specific versus general framework responses. Distortions due to weaker binding states could explain the slight increase in Zr–Zr distance upon introduction of DMMP vapor. Toward the end of the DMMP dosing step, the Zr–Zr distance re-contracted, which is consistent with both increasing strain due to further loading of DMMP and with relaxations due to increasing bidentate states. That the Zr–Zr distance continues to contract during He flow in step V, despite the decrease in pore content, suggests that the increasing amount of bidentate binding may be more important. Re-expansion on heating may suggest changes or competing effects in the response of the DMMP or MMPA-bound clusters to changes in the strain as unreacted DMMP is further removed or to heating. There may also be complicating effects from clusters under-coordinated by more than one BPDC ligand. Overall, further experiments could be useful to better separate these different effects, possibly comparing the temperature-dependence of frameworks with different levels of ligand vacancies, sorbed gases, and types of binding states. Regardless, the Zr–Zr distances observed are consistently larger than the distances assigned to P–Zr pairs, giving further confidence that these intensities do not come from the Zr–Zr distortions.

## Discussion

Here, we have presented a method for obtaining a direct atomic-scale picture of what happens to a MOF structure over an unprecedentedly broad length scale. UiO-67 was found to rapidly adsorb and decompose DMMP accompanied by an ~1.14% increase in cell volume. Gas absorbed in the pore bulk appears to prefer occupying smaller tetrahedral pores, likely due to stronger confinement effects^59^. On flowing He gas at RT, the framework re-contracted due to the exchange of a small amount of DMMP in the pore bulk for He. However, the majority of DMMP remained within the framework over the timescale of the step. Increasing the temperature above ~40 °C resulted in further removal of DMMP. It is possible that the noted NTE behavior of the activated framework can help squeeze out remaining, unreacted DMMP at higher temperatures. Product MMPA bound to the Zr_6_ cores was observed to remain upon re-activation, and the binding states were observed to relax to stronger binding states over the course of the experiment. Similar to those previously observed^11^, these remaining bound states are found to have an effect on the cluster structure as well as an expansive effect on the overall framework. Unlike the previous study, we did not observe a significant decomposition of the framework on reheating.

The study of the gas uptake, reaction, and accompanying structural response in MOFs is a challenging endeavor due to the multiple, correlating thermal and chemical effects over different length scales. We have shown that in situ total scattering and PDF analysis, assisted by DFT simulations, can allow for simultaneous tracking of the expansion/contraction of the lattice, gas uptake and decomposition, and structural effects on the associated clusters. Furthermore, this method can be of significant benefit for studying more defected or disordered MOFs, as the assumptions of Bragg analysis are not limiting in real space. The ability to quantify the changes in various structural components in real-time and under real reaction conditions makes this technique a powerful complement to other multimodal approaches for verifying proposed mechanisms. The work done here suggests that the information obtained from the PDFs alone may be sufficient to disqualify incorrect models, and further constrain potential mechanisms in question. This could help reduce the total number of separate experiments needed for a given system.

As linker vacancies are required for open reactive sites, further studies using in situ PDF analysis of increasingly defective MOFs may yield higher sensitivity to the interactions between CWAs or other guest compounds and the clusters. This could help in understanding the effects of amount or type of defects present in the MOF, thereby enabling syntheses to be designed for optimal activity. This study can provide a blueprint for future studies of a large class of materials, including but not limited to MOFs, to gain an atomic-level understanding of binding and reaction of chemical warfare agents (CWAs) impacting the development of novel filtration media and other protective materials.

## Methods

### Material preparation

The preparation of UiO-67 was modified from the literature^62^. Typically, biphenyl-4,4′-dicarboxylic acid (H_2_BPDC) and ZrCl_4_ (0.64 mmol) were added into DMF (18 mL) and stirred for 30 min. The resulting mixture was transferred to an autoclave and heated at 80 °C for 24 h. White crystalline powders were collected by centrifugation and washed extensively with DMF and anhydrous acetone. Obtained materials were further dried under vacuum at 90 °C for 8 h.

### X-ray total scattering measurements

Total scattering measurements were carried out using the high energy X-ray Powder Diffraction beamline 28-ID-2 (XPD), National Synchrotron Light Source II (NSLS-II), Brookhaven National Laboratory. X-ray scattering data were collected in rapid acquisition mode^63^, using a large-area 2D PerkinElmer detector (2048 × 2048 pixels, 200 × 200 μm^2^ each) with a sample-to-detector distance of ~212 mm. The incident energy of the X-rays was 66.41 keV (*λ* = 0.1867 Å). Powdered UiO-67 was loaded into a 1 mm ID polyimide capillary gas flow cell. Both ends of the capillary were loaded with quartz wool to keep the sample in place, while allowing gas to flow through. The capillary was attached to the flow cell with Swagelok fittings and graphite ferrules. The input of the cell was connected to a gas inlet system that carried He and DMMP vapor/He mixture to the cell. Gas flowed at a rate of 10 mL/min using a mass flow controller. During the DMMP dosing stage, He gas flowed through the saturator filled with DMMP liquid kept at a temperature of ~40 °C. The temperature of the sample was controlled using an Oxford Cryostream. The experiment involved various stages as follows^10^: (I) Unprocessed UiO-67 was first measured at RT (30 min); (II) Activation: starting material was heated to 80 °C under He and measured for 3 h in 5 min increments, then cooled back to RT and measured again (30 min); (III) Reheating: the activated UiO-67 was then reheated under He and measured from RT to 80 °C in 5 °C steps; (IV) DMMP flow: DMMP vapor/He mixture was introduced to the flow cell and measured at RT for 7.3 h in 5 min increments; (V) He flow: DMMP vapor was cut off, He continued, and measured at RT for 4 h in 5 min increments; (VI) Temperature ramp: the sample was heated from RT to 80 °C in 1 °C steps under He and measured for 5 min at each step; (VII) Re-activation: sample was held at 80 °C for 2 h under He and measured in 5 min increments. Longer 30 min scans were measured between each step. Ni was measured at room temperature (RT) as a standard for calibration, and the empty flow cell was measured as a background and subtracted.

### Data reduction

Calibration was performed, and the raw 2D intensities were corrected for polarization and azimuthally integrated and converted to 1D intensity versus *Q* (*Q* = 4*π*sinθ*/λ*, is the magnitude of the scattering momentum transfer for elastic scattering, with 2*θ* scattering angle) using pyFAI^64^. Further correction and normalization of the integrated 1D diffraction intensities, and background subtraction, were carried out to obtain the total scattering structure function, *F*(*Q*), which was Fourier transformed to obtain the pair distribution function (PDF), *G*(*r*) using the reduction methods of PDFgetX3^65–67^ within xPDFsuite^68^. The minimum *Q*_min_ and maximum *Q*_max_ values used in the Fourier transform of the total scattering data were 0.3–24.0 Å^−1^. Real-space PDF refinements used PDFgui^69^. Other refinements were performed using home-written codes in Python using functionality from Diffpy-CMI^70^. See text in Supplementary Sections 1 and 2 and Supplementary Fig. 1 for more details.

### Rietveld refinement

The structure published by Ko et al.^50^ was converted to *F432* symmetry and used as a starting point for Rietveld refinements performed using TOPAS v6^71^. Refinement was performed over a range of 0.5–20° 2*θ* using a global scale factor, Gaussian peak broadening term, LP-factor equal to 90 for synchrotron radiation, and a background function consisting of a Chebychev polynomial of 17th order and a 1/*X* background function to account for increased background intensities at lower angles. Zr and O sites were refined by *F432* symmetry to the diffraction pattern of the empty framework. Separate *Biso* values for Zr, O, and C atoms and separate occupancy factors for the carboxylic O atoms and C atoms in the linker were applied for the empty framework, though the carboxylic O atoms refined to 1.0 (full occupancy), indicating the presence of other species at sites left open by linker vacancies. The mentioned site positions, occupancy factors, and *Biso* values were subsequently fixed. The pseudo-atom positions and *Biso* (=156 Å^2^) were determined as discussed above, by refinement at maximum gas loading and then also fixed. Finally, the lattice parameter, occupancies of the pseudo-atoms, scale, and background were refined for all datasets. The structure model used with pseudo-atom sites, from refinement at maximum gas loading, is provided in Supplementary Data 1.

### Density functional theory calculations

All calculations were carried out with the VASP 5.4.1 suite^72^ and considered the PBE functional^73^. Projected-augmented-wave pseudopotentials^74^ were used in combination with a plane-wave basis with a 540 eV cutoff throughout. The Brillouin zone sampling was constrained to the Γ point. A 0.02 eV/Å maximum force per atom cutoff was used in the atomic relaxation. The computational model considered the UiO-66^75^ cubic unit cell (Zr_24_O_128_C_192_H_112_) with a missing linker defect. In the structure that models the MOF prior to DMMP exposure, the under-coordinated Zr atoms were saturated with hydroxo and aqua ligands, as determined experimentally. DMMP replaced an aqua ligand upon binding and the reaction proceeded as detailed in earlier work to lead to methyl methylphosphonic acid (MMPA)^60,76^.

## Supplementary information

Supplementary Information

Description of Additional Supplementary Files

Supplementary Data 1

## Data Availability

The data supporting the findings of this study are available within the article and its Supplementary Information files. Additional information and figures are provided in the Supplementary Information, and DFT-derived and experimental model structures are provided in Supplementary Data 1. Other data are available from the authors upon request.
