# Supplementary material for: Atomic resolution tracking of nerve-agent simulant decomposition and host metal–organic framework response in real space
Source: Commun Chem. 2021 Jan 4;4:2. doi: 10.1038/s42004-020-00439-1 (PMC9814582; doi:10.1038/s42004-020-00439-1)
Supplement: Supplementary file 1 — Supplementary Information [file 42004_2020_439_MOESM1_ESM.pdf]

Supplementary Information:

Atomic resolution tracking of nerve-agent simulant decomposition and host metal−organic framework response in real space

Maxwell W. Terban[a], Sanjit K. Ghose[b,*], Anna M. Plonka[c], Diego Troya[d], Pavol Juhás[e], Robert E. Dinnebier[a], John J. Mahle[f], Wesley O. Gordon[f], Anatoly I. Frenkel[c,g,*]

[a]Max Planck Institute for Solid State Research, Heisenbergstr. 1, 70569 Stuttgart, Germany

[b]National Synchrotron Light Source II, Brookhaven National Laboratory, Upton, New York 11973, United States [c]Department of Materials Science and Chemical Engineering, Stony Brook University, Stony Brook, New York 11794, United States

[d]Department of Chemistry, Virginia Tech, Blacksburg, Virginia 24061, United States

[e]Computational Science Initiative, Brookhaven National Laboratory, Upton, New York 11973, United States

[f]U.S. Army Combat Capabilities Development Command Chemical Biological Center, Aberdeen Proving Ground, MD 21010, United States

[g]Chemistry Division, Brookhaven National Laboratory, Upton, New York, NY 11973, United States

Table of Contents

1. Pair distribution function analysis……………………………………………………………....…….2

2. Effects of gas composition on PDF data reduction …….………………………….………………...…….3

3. Diffraction intensities and lattice parameters………………………..………………..…...….4

4. DFT models…………………………………………………………………………………….…...….7

5. Difference PDFs referenced to activated sample………………………………...……...…….9

6. Structure-independent model………………….…………………………………...…...….10

7. Structure-dependent model……………………………………………………….………...….13

8. Zr-Zr distances from DFT calculations.………………………………………….………...….16

9. Thermal analysis…………………..……………………………………….………...….16

Supplementary References………………………………………………………………………….17

Supplementary Section 1: Pair distribution function analysis

Total scattering measurements over a wide range of momentum transfer and with good statistics are required to obtain suitable PDFs for structure analysis. The coherent diffraction intensities are normalized by the form factors of the sample composition to obtain the total scattering structure function $S(Q)$, which is then Fourier transformed by

$$G(r) = \frac{2}{\pi}\int_{Q_{min}}^{Q_{max}} Q[S(Q) - 1]sin(Qr)dQ \tag{1}$$

$F(Q) = Q[S(Q) - 1]$ is the reduced total scattering structure function, and $G(r)$ is the PDF.[1,2] In practice, values of Q_{min} and Q_{max} are determined by the experimental setup, and Q_{max} is often reduced below the experimental maximum to reduce the effects of low signal-to-noise in the high-Q region on the Fourier transformation. The PDF gives the scaled probability of finding two atoms in a material a distance r apart and is relative to the density of atom pairs in the material. For a macroscopic scatterer, $G(r)$ is calculated from a known structure model according to

$$G(r) = 4\pi r[\rho(r) - \rho_0], \tag{2}$$

$$\rho(r) = \frac{1}{4\pi r^2 N}\sum_i \sum_{j\neq i} \frac{f_i f_j}{\langle f\rangle^2}\delta(r - r_{ij}). \tag{3}$$

Here, ρ_0 is the average number density of the material and ρ(r) is the local atomic pair density, which is the mean weighted density of neighbor atoms at distance r from an atom at the origin. The sums in $\rho(r)$ run over all atoms in the sample, f_i is the scattering factor of atom i, $\langle f\rangle$ is the average scattering factor and r_{ij} is the distance between atoms i and j. In this study, Eqs. 2 and 3 were used to fit the PDF generated from a structure model to the experimental PDFs in using the program PDFgui.[3] The delta functions in Eq. 3 were Gaussian-broadened to account for atom displacements and the finite Q_{range} of the experiment. PDF modeling was performed by adjusting the lattice parameters, atomic positions where allowed by symmetry**,** atomic displacement parameters (ADPs), correlated motion of neighboring atoms δ2,[4] domain size (*spdiameter*), a global scale factor, and phase-specific scale factors for multiphase fits. The resolution parameters due to Q-space resolution, Q_{damp} = 0.0327 Å^{-1}, and due to variable Q-resolution and high-Q noise, Q_{broad} = 0.01067 Å^{-1},[5,6] were determined from experimental data of powder standard Ni and used for further fitting. The refinements were run by minimizing R_w, between the dataset and the model with set of refined parameters P, calculated as

$$R_w = \sqrt{\left(\sum_{i=1}^{n}[G_{obs}(r_i) - G_{calc}(r_i, P)]^2\right)\Big/\left(\sum_{i=1}^{n} G_{obs}(r_i)^2\right)}. \tag{4}$$

Supplementary Section 2: Effects of gas composition on PDF data reduction

The starting composition used in processing the PDF data was $Zr_6O_{36}C_{84}H_{60}$, representing the composition of an empty framework. Since we were not able to accurately measure the changing composition of the sample during gas loading, we investigated the effects of possible changes in composition on the resulting PDF data. To estimate an upper bound for DMMP loading, we considered that the density of liquid DMMP ($C_3H_9O_3P$) is 1.145 g/cm^3 at 25 °C (according to Sigma Aldrich CSDS), molar mass is 124.08 g/mol, and volume available to DMMP in pristine UiO-67 is 8312.67 Å^3 or with no linkers at all 11,119.20 Å^3 (as calculated using Mercury). For 50% linker vacancy (this is likely an overestimate), we would get ~65 molecules per unit cell, and therefore (4 clusters per cell) 16.25 molecules stoichiometrically, so $Zr_6O_{36}C_{84}H_{60}$•16.25($C_3H_9O_3P$). In Fig. S1 we show the results using this composition versus the composition of the framework alone.

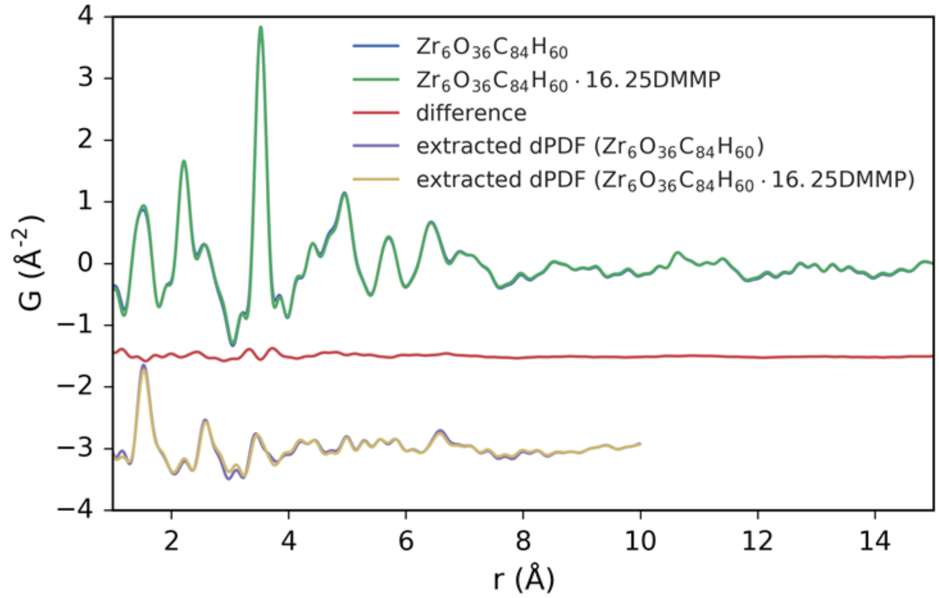

Supplementary Figure 1: Effect of chemical composition on the PDFs. Comparison of the PDF from the last data point of the DMMP loading step calculated with compositions of "$Zr_6O_{36}C_{84}H_{60}$" (blue) or "$Zr_6O_{36}C_{84}H_{60}$•16.25($C_3H_9O_3P$)" (green). The difference (red) shows the small effect. Below these curves, we also show the extracted (1-10 Å) dPDFs with respect to the empty framework PDF with composition "$Zr_6O_{36}C_{84}H_{60}$" in both cases – also showing negligible changes.

Since this is an overestimate and most of the interesting trends observed are after DMMP dosing, the effects of changing composition will be even less than demonstrated above. Thus, we found that using $Zr_6O_{36}C_{84}H_{60}$ consistently for the composition was a suitable assumption for our analysis. It would be important to more accurately account for this in cases where loaded species contain heavy metal atoms, and future implementations could be made even more accurate by incorporating this detail. Furthermore, this would be necessary if one wanted to try to measure accurate coordination #s or perform RMC modeling.

Supplementary Section 3: Diffraction intensities and lattice parameters

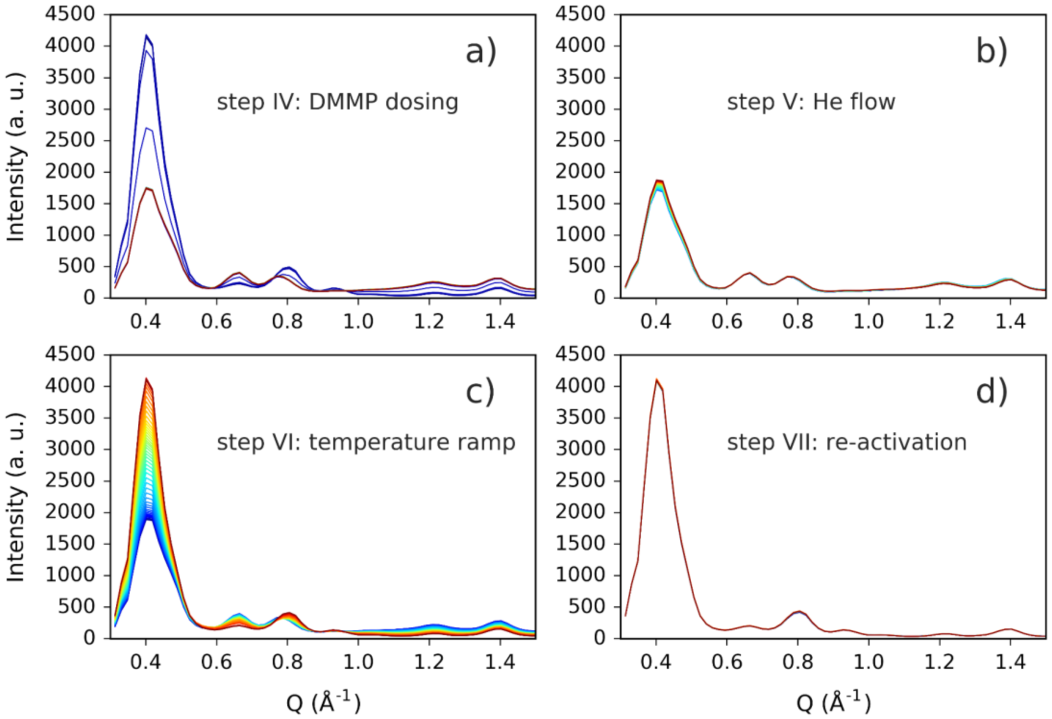

Supplementary Figure 2: Sequential diffraction patterns shown for different steps of the experimental procedure. The patterns are plotted from blue (start) to end (red) of each respective step. Peaks are broadened due to the high energy and short sample-to-detector distance of the experiment. The first large peak corresponds to the 111 and 200 Bragg reflections of the cubic UiO-67 structure.

Supplementary Table 1. Lattice parameters of UiO-67 determined by reciprocal (Rietveld) and real space (PDF) refinements for different steps during the activation and heating processes. Errors given are as reported in the refinement software.

		Before activation (starting material)	During Activation (end of step II)	After Activation	Re-heated (end of step III)
Temperature (°C)		27	27	80	27
a (Å)	Rietveld	26.849(4)	26.841(5)	26.854(4)	26.844(5)
	PDF	26.852(30)	26.846(30)	26.852(30)	26.847(30)
	Average	26.851(15)	26.844(15)	26.853(15)	26.846(15)

To support the observed changes in relative diffraction peak intensities, the effects of ordered versus disordered pore content were simulated from corresponding models. To demonstrate this, we have simulated three diffraction patterns: 1. an empty framework, 2. the framework with 64 atoms (15 e^- each) placed at specific locations distributed within the pores and same Biso as framework atoms (ordered DMMP), and 3. the framework with same number of e^- centered at 50% octahedral and 50% tetrahedral sites with Biso = 400× that of the framework atoms (disordered DMMP).

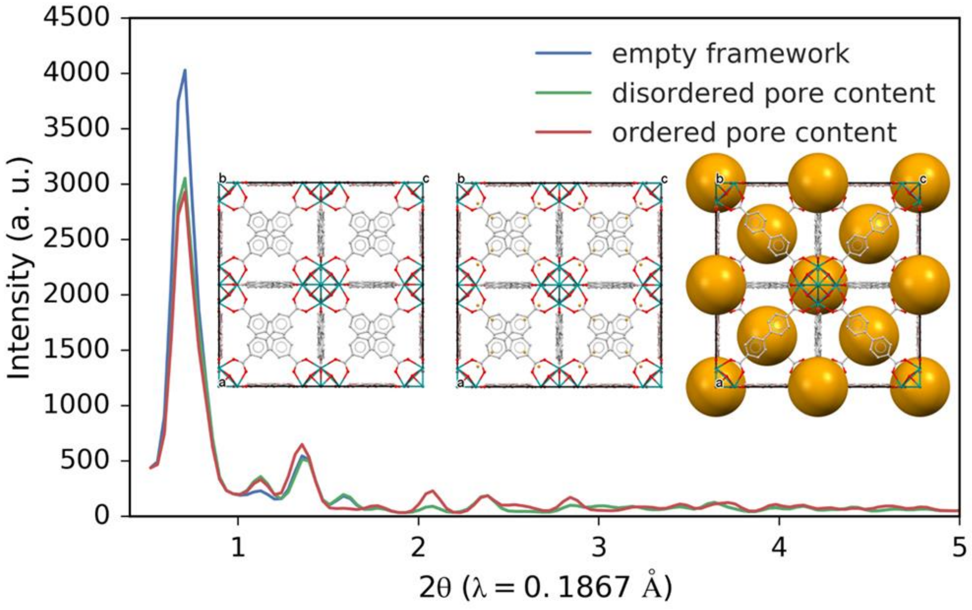

Supplementary Figure 3: Simulated diffraction patterns resulting from empty framework, and the framework with ordered or disordered pore content. The patterns show a similar reduction in first peak intensity for same amount of pore content, regardless of state of ordering. The inset shows the three structure models used to simulate diffraction patterns: empty framework, framework with ordered pore content, and framework with disordered pore content from left to right.

The main difference is that the ordered pore content contributes distinct changes at higher angles while the disordered content does not. Therefore, while it is difficult to quantify the absolute number of electrons in the pore due to smearing effects on the pseudo-atoms in our model, this modulation in intensities is still a good measure of whether molecules are present or leaving in the bulk of the pore with respect to the starting framework, regardless of state of ordering.

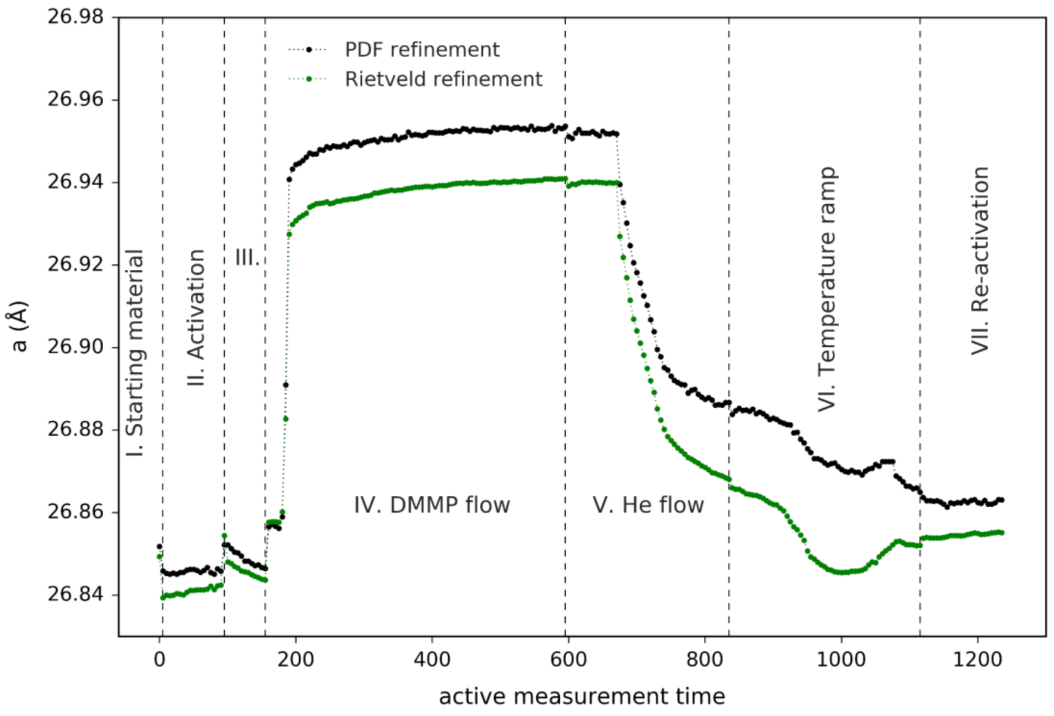

Supplementary Figure 4: Rietveld- versus PDF-derived lattice parameters. Comparison of lattice parameters obtained from real space PDF (black) and reciprocal space Rietveld (green) refinements. The trends agree very well. There is a slight systematic shortening of the Rietveld refined lattice parameters. This could be attributed to the very broad profile of the reciprocal space peaks, due to the optimization of the total scattering measurement for real space analysis. An alternative consideration is that since the PDF refinements were performed up to 35 Å, they basically measure the lattice parameter according to the average intercluster spacing. Therefore, the lattice parameter obtained from Rietveld refinement could be slightly more contracted due to the effect of cluster vacancies on the bulk average lattice parameter.

Supplementary Section 4: DFT models

Supplementary Scheme 1: Structures from DFT calculations. Files are available for download.

1. DMMP.xyz --	DMMP	
2. pristine.cif --	Zr—O<br>Zr—O ＞ C— linker —C ＜	O—Zr<br>O—Zr
3. missing-linker-oh-h2o-oh-h2o.cif ---------------	Zr—OH_2<br>Zr—OH	H_2O—Zr<br>HO—Zr
4. missing-linker-oh-oh.cif ---------------------------	Zr<br>Zr—OH	—Zr<br>HO—Zr
5. missing-linker-oh-dmmp-oh.cif ------------------	Zr—DMMP<br>Zr—OH	—Zr<br>HO—Zr
6. missing-linker-oh-dmmp-oh-h2o.cif ------------	Zr—DMMP<br>Zr—OH	H_2O—Zr<br>HO—Zr
7. missing-linker-oh-mmpa-monodentate-oh.cif	Zr—MMPA<br>Zr—OH	—Zr<br>HO—Zr
8. missing-linker-oh-mmpa-oh.cif ------------------	Zr<br>Zr ＞ MMPA	—Zr<br>HO—Zr
9. missing-linker-oh-mmpa-oh-h2o.cif ------------	Zr<br>Zr ＞ MMPA	H_2O—Zr<br>HO—Zr

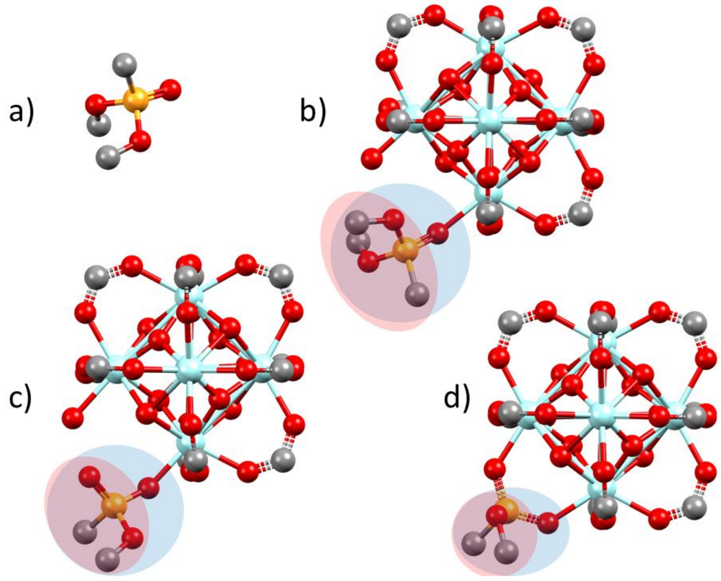

Supplementary Figure 5: Extracted portions of the DFT calculated structures used for dPDF simulations. (a) isolated DMMP molecule as in file 1, **DMMP.xyz**; **(b)** bound DMMP as in **missing-linker-oh-dmmp-oh-h2o_cluster.cif**; **(c)** monodentate bound MMPA as in **missing-linker-oh-mmpa-monodentate-oh.cif**; **(d)** bidentate bound MMPA as in **missing-linker-oh-mmpa-oh-h2o.cif**. Blue transparent ellipses highlight the atoms use for calculating the dPDFs with bridging oxygen and red transparent ellipses highlight the atoms used for the dPDFs without the bridging oxygen. We did not identify substantial differences in the dPDFs from DFT structures with or without an aqua ligand on the opposing cluster. See section on **Structure-dependent model** below for how the dPDFs were simulated.

Supplementary Section 5: Difference PDFs referenced to activated sample

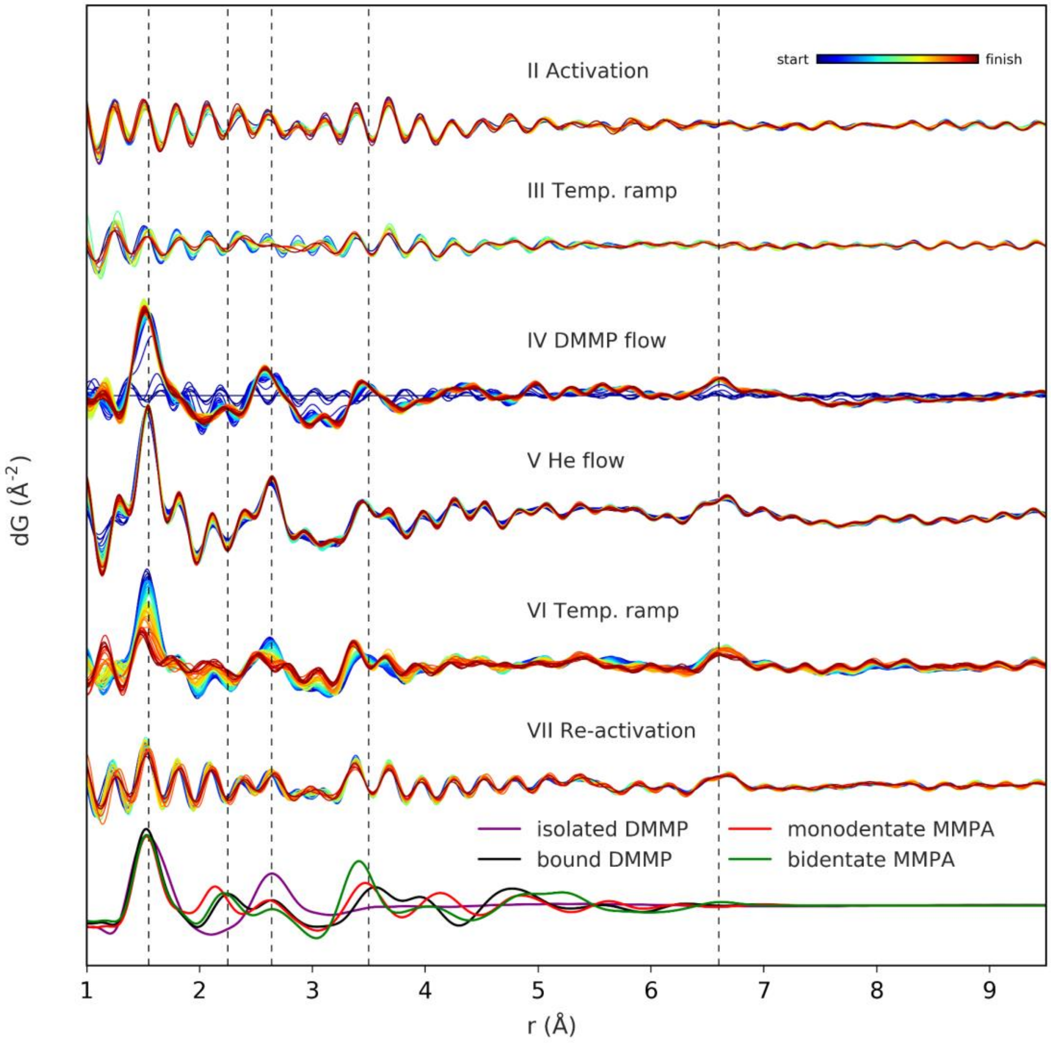

Supplementary Figure 6: The dPDFs for each experimental step. These dPDFs show the differential change in pair distance distribution with respect to the PDF of the activated sample, and with each step offset for comparison. Signals observed in the dPDFs at any step represent the changes in the structural state at that point with respect to the activated sample. The simulated PDF for an isolated molecule of DMMP and the *d*PDFs of DMMP and MMPA binding states are plotted for reference. Here, in comparison to **Figure 6** in the main text, we can see signals remaining in the dPDFs in step VI, where intensities assigned to 1st NN correlations in MMPA and the P-Zr distance can be observed. Some intensity at these positions can even be observed in step VII, which were picked up by both models and explain the preference of the data for the bidentate MMPA state at the end of the experiment.

Supplementary Section 6: Structure-independent model

As described in the paper, the structure-independent model consisted of a reference PDF, $G_{ref}(r)$ which was fitted to the target PDF by a linear scaling and stretching transformations with scale factor A_0 and stretch factor A_1. The changes in density modulation by gas filling in the pores is primarily encoded in the changing intensities in the 111 and 200 Bragg peaks, which leads to a flattening of the pore-framework oscillation in real space. This change can be roughly accounted for by a damped sine wave added to the model, with a scale factor B_0 that is negative for pores that are more filled than the reference PDF, a frequency term B_1 that corresponds to the position of the Bragg peaks in reciprocal space (i.e. the frequency of the density modulation), and a damping term B_2 that corresponds to the breadth of the associated Bragg peaks. Finally, we add three Gaussian peaks which correspond to the three distinct features observed in the dPDFs. All the components are added together to form the total synthesized PDF, $G_{synth}(r)$, as

$$G_{synth}(r) = A_0 G_{ref}(r \times A_1) + B_0 \sin(B_1 r)\, e^{-B_2 r} + Peaks(r), \tag{5}$$

where

$$Peaks(r) = \sum_{i=1}^{3} \sqrt{\ln(2)/\pi}\ (C_i/E_i) \exp\left(-\ln(2)\left(\frac{r - D_i}{E_i}\right)^2\right). \tag{6}$$

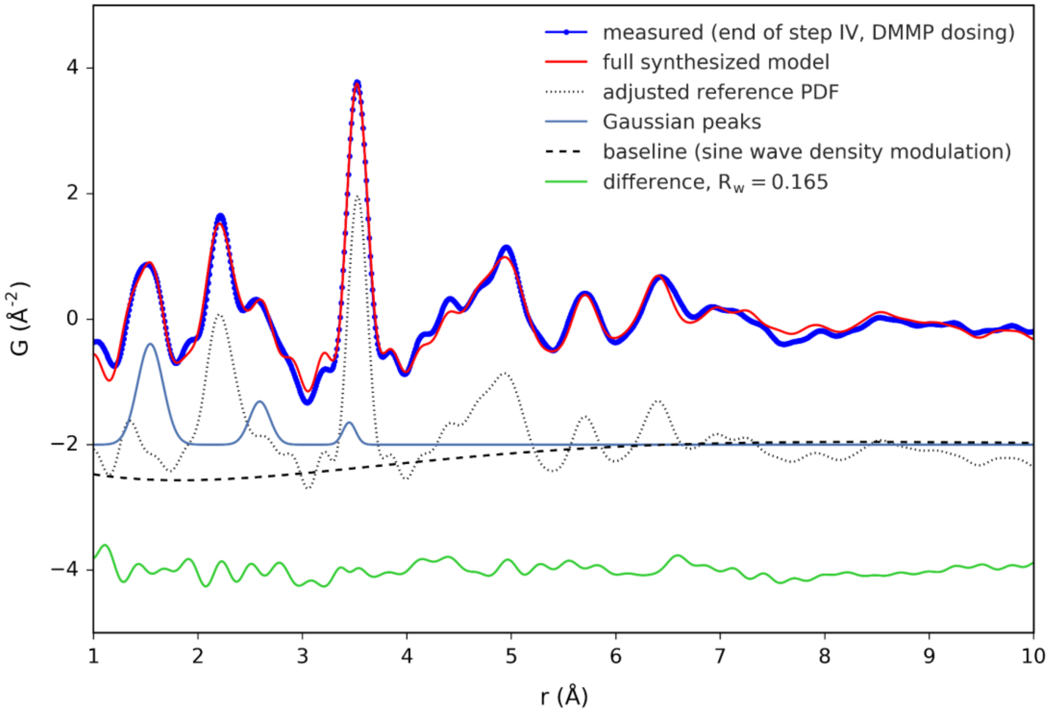

Supplementary Figure 7: Structure-independent fit example. Example of the fit and associated components from refinement of the structure-independent model to the PDF measured at the end of the DMMP dosing step.

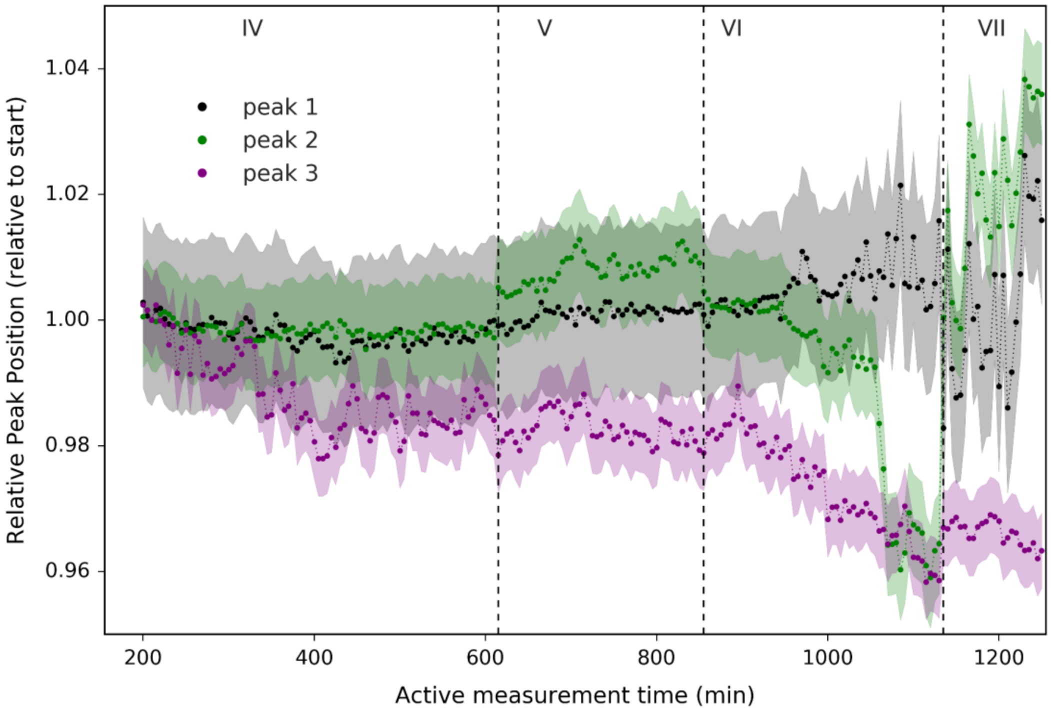

Supplementary Figure 8: Trends in relative peak positions. Every refined peak position here is divided by the position of the peak on first appearance. We see that peak 1, which corresponds to the 1st NN shell in DMMP/MMPA does not change position much throughout the whole experiment. This is expected, because the intramolecular bond distances only change a minute amount whether the molecule is isolated or bound. Peak 2, which corresponds primarily to the 2nd NN shell in the unbound DMMP molecule also does not change position much. Toward the end of heating step VI, the peak appears to change drastically, but this is because the position becomes unreliable when the intensity becomes essentially zero (i.e. free DMMP has mostly left by this point) Figure 8. Peak three is assigned to P-Zr distances, and we can see here that the this peak position decreases slowly throughout the whole process, and more rapidly after heating begins. This can either indicate that bound states relax toward bidentate MMPA, or that unbound coordinating molecules are removed on heating, which could increase the ability to resolve only the distance of the strongly bound states.

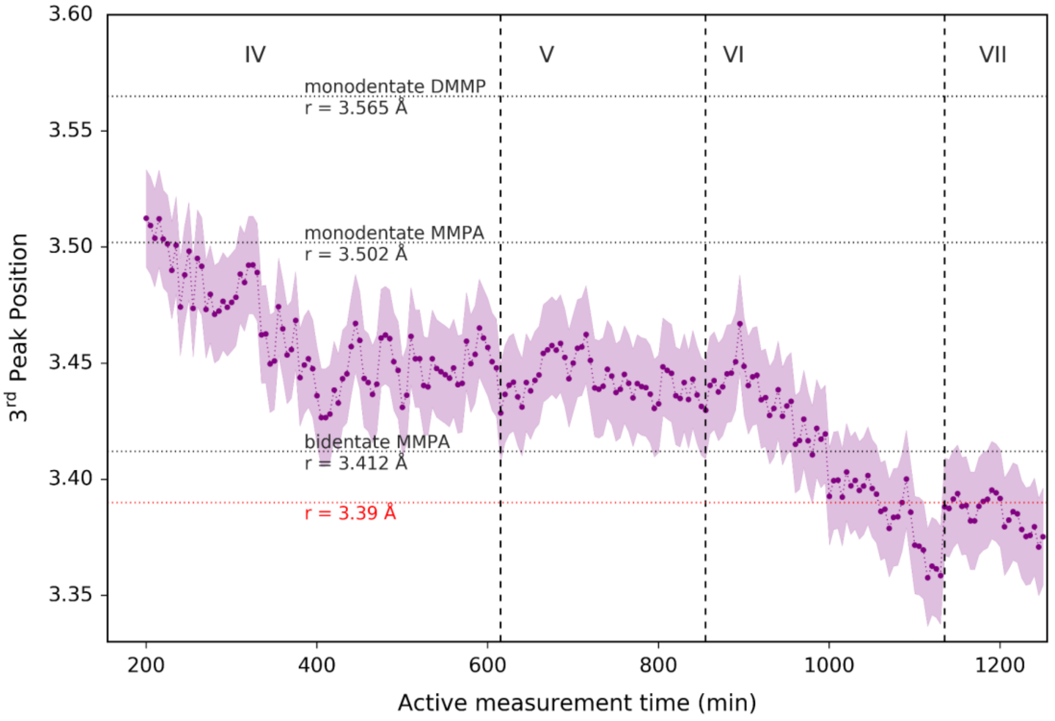

Supplementary Figure 9: Actual refined distances for peak 3. By comparison to the DFT structure distances for P-Zr, this peak is best indexed by bound MMPA states, and decreases to good agreement with bidentate MMPA at the end.

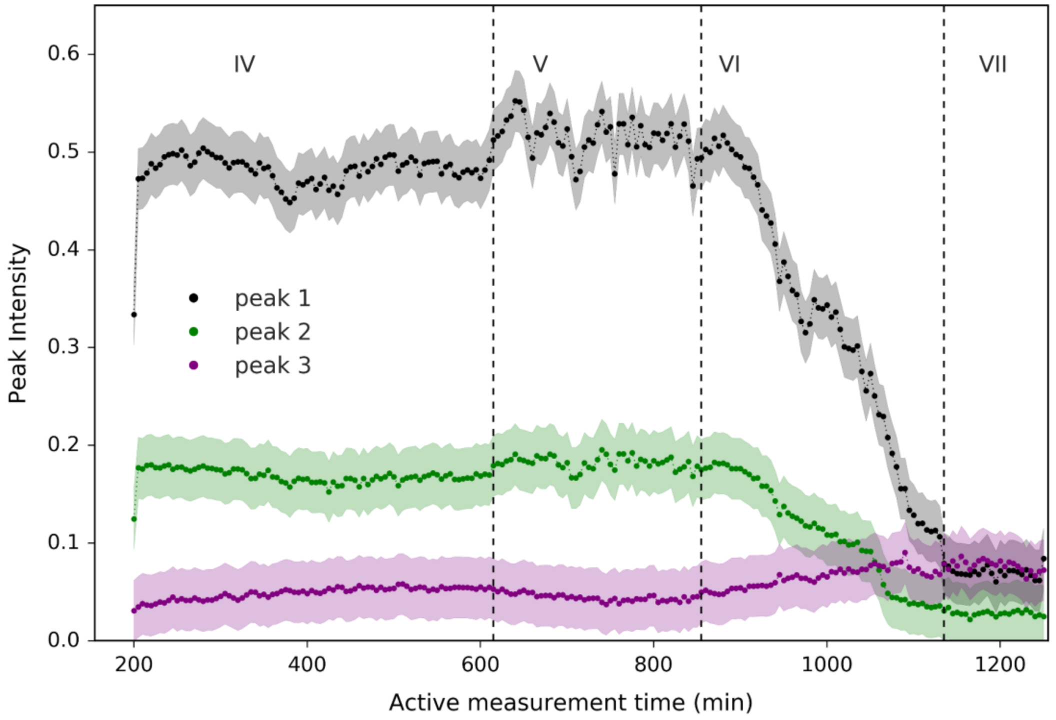

Supplementary Figure 10: Trends in refined peak intensities from the structure independent model. Peaks 1 and 2 show similar trends and relative intensities to the free DMMP signal. Peak three is relatively low throughout, but never goes away, indicating that bound states do not leave on heating or re-activation. At the end Peak 1 intensity decreases significantly as unreacted DMMP leaves, however, some intensity remains which should then correspond to the 1st NN shell in the MMPA molecules remaining bound to the cluster.

Supplementary Section 7: Structure-dependent model

The structure dependent model used shares the same reference PDF, G_{ref}, and density modulation (damped sine wave) components as the structure-independent model. Here, the dPDF component, guest (free or bound) signals, are now simulated from the DFT models and added to the previous model instead of the Gaussian peaks as

$$G_{synth}(r) = A_0 G_{ref}(r \times A_1) + B_0 \sin(B_1 r)\, e^{-B_2 r} + G_{sim}(r). \tag{7}$$

$G_{sim}(r)$ is the sum of the simulated signals, which are calculated as the sine Fourier transformation of the reduced total scattering structure functions by

$$G_{sim}(r) = \sum_{1,2,3}^{binding\ states} \frac{2}{\pi} \int_{Q_{min}=1.70}^{Q_{max}=24.0} F_i(Q)\, sin(Qr) dQ, \tag{8}$$

using the experimental Q_{max} and a finite Q_{min} to remove the molecular small angle scattering region which is not observed for the bulk material, or measured experimentally. Only three binding states were considered at a time, including isolated DMMP, bound DMMP, monodentate MMPA, and/or bidentate MMPA (calculated from models shown in **Figure 3**). Free DMMP was always considered, along with two of the latter three in different trials. We also considered trials with the bridging oxygen considered as part of the cluster rather than the molecule, as we did not observe any significant signal at ~2.25 Å, which may be because there is already a significant amount of Zr-O pairs in the framework, or because we may be replacing remaining aqua ligands in the starting state. Finally, the reduced structure functions were calculated using the Debye equation as

$$F(Q) = Q[S(Q) - 1] = \frac{1}{N\langle f(Q)\rangle^2} \sum_i \sum_{i \neq j} f_i^*(Q) f_j(Q) \frac{\sin(Q r_{ij})}{r_{ij}} \exp\left(-\frac{1}{2}\sigma_{ij}^2 Q^2\right), \tag{9}$$

where σ_{ij}^2 contains the terms for thermal motion and correlated motion of nearby atoms. Here we have fixed the ADPs for all atoms $B_{iso} = 1.0$ Å^{-1} and correlated motion term $\delta_2 = 1.7$ Å^{-2}, which sharpens short distance peaks with a $1/r^2$ dependence (these values were chosen heuristically for these types of materials and fixed (different values were tested and did not have a large impact on the results). Calculations were performed using Diffpy-CMI.[7]

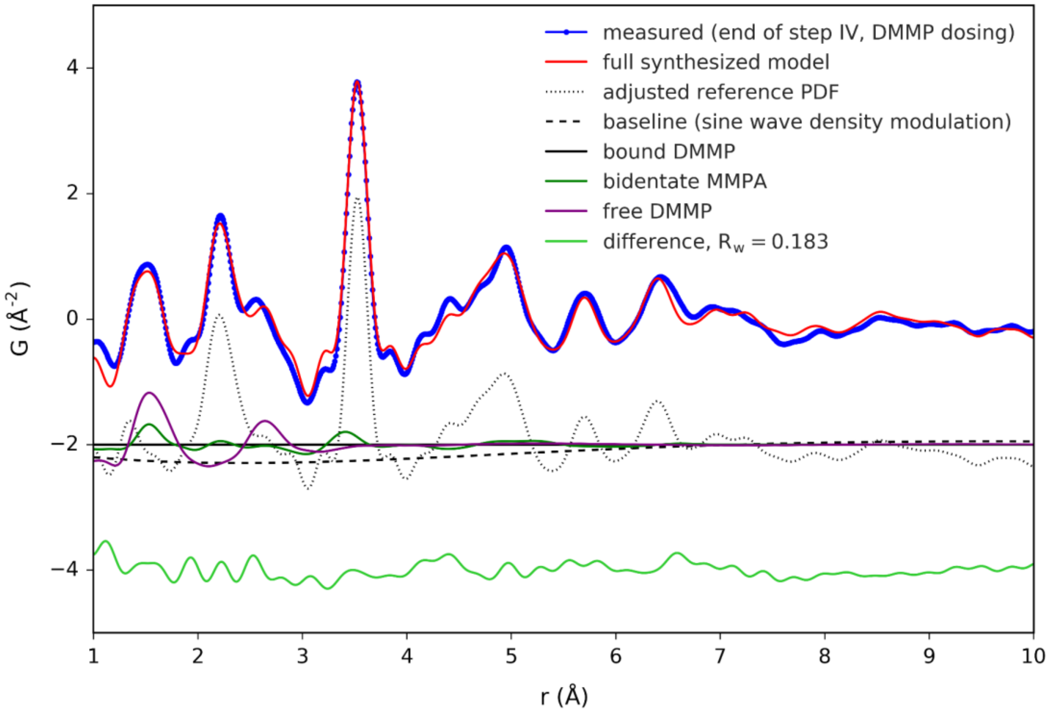

Supplementary Figure 11: Structure-dependent fit example 1. Example of the fit and associated components from refinement of the structure-dependent model to the PDF measured at the end of the DMMP dosing step.

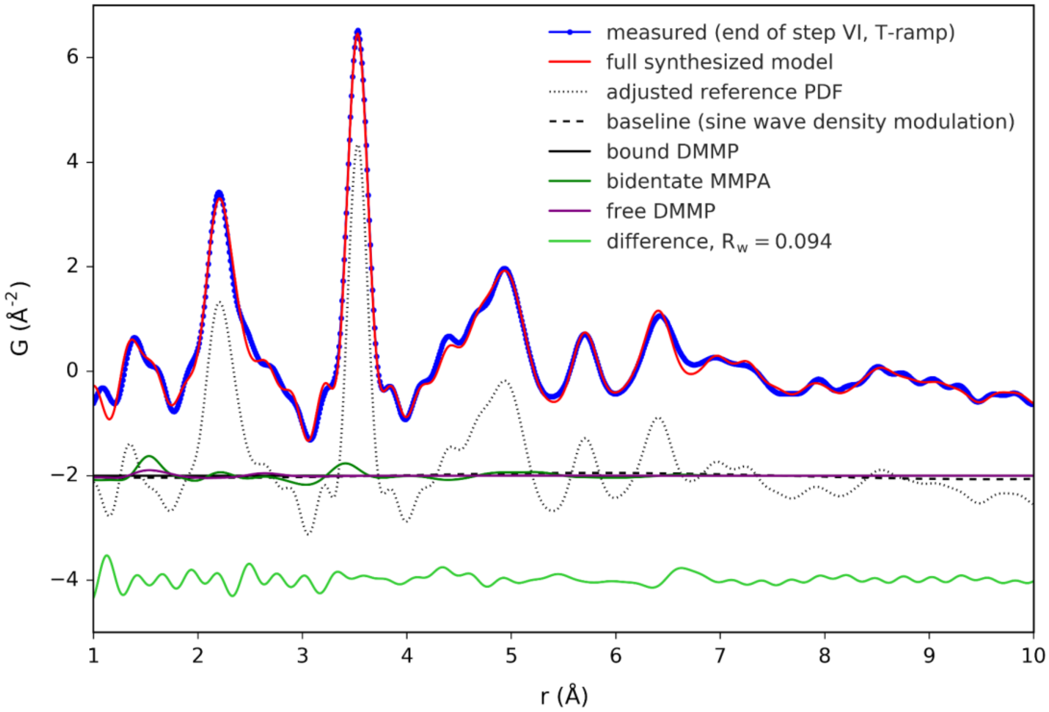

Supplementary Figure 12: Structure-dependent fit example 2. Example of the fit and associated components from refinement of the structure-dependent model to the PDF measured at the end of the temperature ramping step.

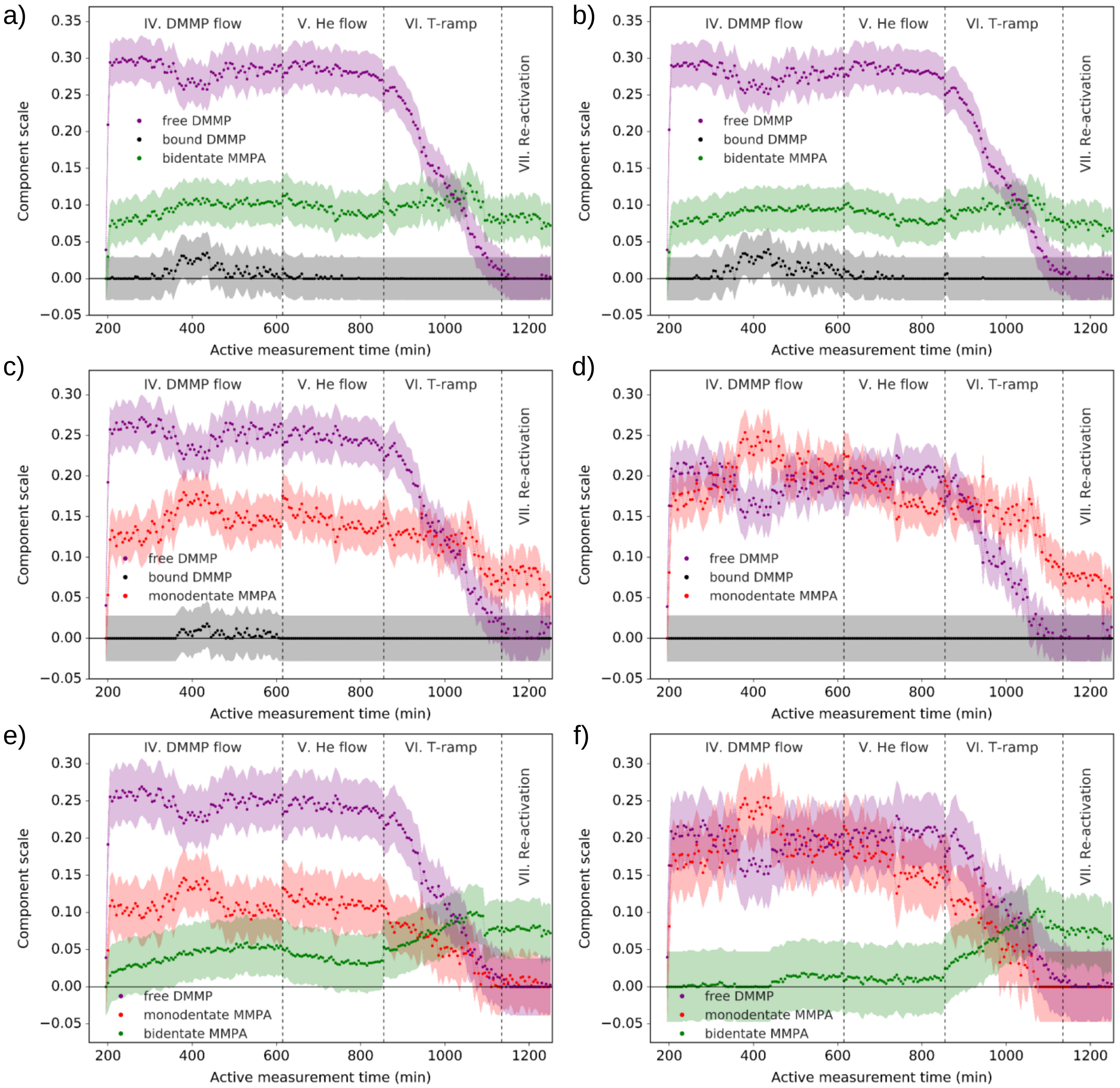

Supplementary Figure 13: Example of different combinations of dPDF signals to include in the structure-dependent model. Refinement performed with **(a,c,e)** with bridging oxygen considered as part of the molecule, or **(b,d,f)** with bridging oxygen considered as part of the cluster. Signal from bound DMMP was always found to be negligible. The refinements tended to prefer the dPDF signal of monodentate MMPA versus bidentate MMPA, however the ending always preferred bidentate MMPA. Since the monodentate MMPA signal goes away in either case, we can reasonably assign this signal to either monodentate MMPA that then further relaxes to a bidentate state, or it could also account for unbound, coordinating DMMP, or bound DMMP prior to degradation. Further work on samples that give higher binding is needed to make these determinations for sure.

Supplementary Section 8: Zr-Zr distances from DFT calculations

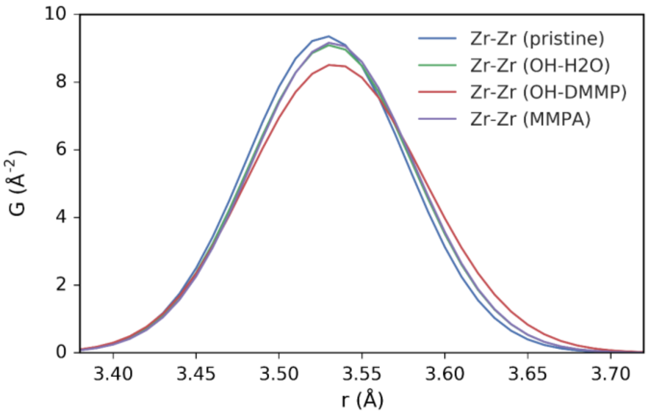

Supplementary Figure 14: Zr-Zr distances from DFT. Comparison of 1st neighbor Zr-Zr distances simulated from DFT relaxed clusters with full linker occupation (blue), or with a single linker vacancy filled by one hydroxyl and one water (green), one hydroxyl and DMMP (red), or bidentate MMPA (purple). The PDFs were calculated with the experimental Q_{max} =24.5 Å^{-1} and *Biso* = 0.1 Å^2, showing that even with very small atomic displacement parameters, the predicted distortions would be too small to observe in our experiment.

Supplementary Section 9: Thermal analysis

A sorbent characterization was performed for DMMP into UiO-67. The adsorption isotherm was measured for DMMP at T = 23 °C, and then the exposed sample was analyzed by TGA. Prior to the experiment, UiO-67 was heated to 80 °C in a box furnace in air for 2 hours, and then immediately sealed in a vial.

The adsorption isotherm was obtained by measuring the adsorbed-phase concentration (loading) as a function of the vapor-phase concentration at constant temperature. The isotherm apparatus is comprised of three subsystems: (1) the vapor-phase concentration control system, (2) the adsorbed-phase measurement system, and (3) the temperature-control system, all controlled by software written in Labview. A set of flow controllers were used to vary the fraction of flow through a liquid-vapor contactor and dry N_2 source. The vapor-liquid equilibrator system consists of four main parts:[8] a liquid reservoir, an aluminum oxide wick, an inlet, and an outlet. The liquid reservoir is designed to accommodate a maximum of 10 ml of liquid and is filled from the inlet port. A cylindrical aluminum oxide tube, located in the reservoir, serves as a wick and absorbs the liquid. The uptake weight was recorded using a Cahn microbalance (Model D-200, Cahn Instruments, Cerritos, CA) until equilibrium was achieved. The feed vapor first passes through a coiled glass tube located within the temperature-controlled, water-jacketed test chamber. A second circulating water bath is used to maintain the desired temperature of the test chamber. After temperature conditioning, the feed vapor is passed to the center tube of the test chamber and over the adsorbent basket. The effluent temperature of the vapor stream is measured in this area using a type T thermocouple where the standard accuracy of ±1.0°C is assumed. Overall design and operation of the gravimetric apparatus used for the adsorption isotherm is described previously.[9,10]

The TGA experiments were conducted using a TA Instruments (New Castle, DE) model SDT Q600. Approximately 5 mg of sample was placed in a tared platinum cup. The sample weight was recorded using a temperature ramp of 10 °C/min with an N_2 flow of 10 ccm.

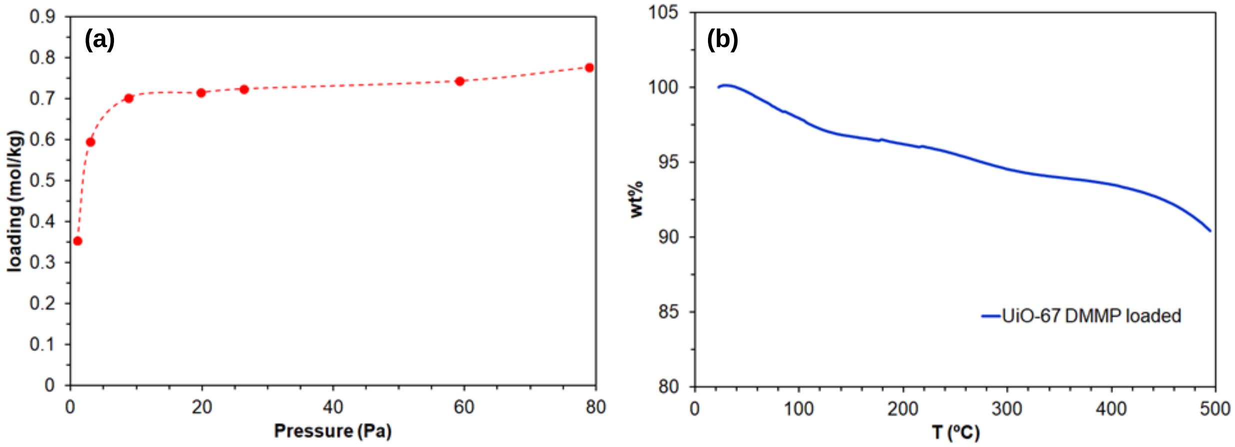

Supplementary Figure 15: Thermogravimetric behavior. (a) DMMP adsorption isotherm (T = 23 °C); the maximum uptake was 96 mg/g. (b) Thermogravimetric analysis curve reporting weight loss versus temperature for UiO-67 with adsorbed DMMP. In the range of 23–80 °C, no two-step weight loss was observed, suggesting continuous desorption of DMMP and/or DMMP break down products. By 120 °C the adsorbed DMMP is removed.

Supplementary References

1 Egami, T. & Billinge, S. J. L. *Underneath the Bragg peaks: structural analysis of complex materials*. (Pergamon Press, Elsevier, 2012).

2 Billinge, S. J. L. Nanometre-scale structure from powder diffraction: total scattering and atomic pair distribution function analysis. *Int. Tabl. Crystallogr.* **H**, 649-672 (2019).

3 Farrow, C. *et al.* PDFfit2 and PDFgui: computer programs for studying nanostructure in crystals. *J. Phys.-Condens. Mat.* **19**, 335219 (2007).

4 Jeong, I.-K., Proffen, T., Mohiuddin-Jacobs, F. & Billinge, S. J. L. Measuring correlated atomic motion using X-ray diffraction. *J. Phys. Chem. A* **103**, 921-924 (1999).

5 Olds, D. *et al.* Precise implications for real-space pair distribution function modeling of effects intrinsic to modern time-of-flight neutron diffractometers. *Acta Cryst. A* **74**, 293-307 (2018).

6 Qiu, X., Božin, E. S., Juhas, P., Proffen, T. & Billinge, S. J. L. Reciprocal-space instrumental effects on the real-space neutron atomic pair distribution function. *J. Appl. Cryst.* **37**, 110-116 (2004).

7 Juhás, P., Farrow, Christopher L., Yang, X., Knox, Kevin R. & Billinge, Simon J. L. Complex modeling: a strategy and software program for combining multiple information sources to solve ill posed structure and nanostructure inverse problems. *Acta Cryst. A* **71**, 562-568 (2015).

8 Butrow, A. B., Buchanan, J. H. & Tevault, D. E. Vapor pressure of organophosphorus nerve agent simulant compounds. *J. Chem. Eng. Data* **54**, 1876-1883 (2009).

9 DeCoste, J. B., Denny, J. M. S., Peterson, G. W., Mahle, J. J. & Cohen, S. M. Enhanced aging properties of HKUST-1 in hydrophobic mixed-matrix membranes for ammonia adsorption. *Chem. Sci.* **7**, 2711-2716 (2016).

10 Tovar, T. M., Mahle, J. J., Knox, C. K. & LeVan, M. D. 110th anniversary: molecular structure effects on mass transfer of C10 hydrocarbons in BPL activated carbon. *Ind. Eng. Chem. Res.* **58**, 15271-15279 (2019).
