# Supplementary material for: Atomic resolution tracking of nerve-agent simulant decomposition and host metal–organic framework response in real space
Source: Commun Chem. 2021 Jan 4;4:2. doi: 10.1038/s42004-020-00439-1 (PMC9814582; doi:10.1038/s42004-020-00439-1)
Supplement: Supplementary file 2 — Description of Additional Supplementary Files [file 42004_2020_439_MOESM2_ESM.pdf]

Description of Additional Supplementary Files

File Name: Supplementary Data 1
Description: DFT-derived and experimental model structures.
